# UV-C irradiation and hermetic storage modulate essential oil profiles and antifungal bioactivity of Apiaceae fruits during long-term storage

**DOI:** 10.1186/s12870-026-09644-x

**Published:** 2026-08-15

**Authors:** Alia Amer, Reham M. Kamel, Aml Shahin, Hend T. E., Mohamad Y. S. Ahmed, Rania A. S., Eman A. Hamed

**Affiliations:** 1 Medicinal and Aromatic Plants Research Department, Horticulture Research Institute, Agricultural Research Center, Giza, Egypt; 2 Agricultural Engineering Research Institute, Agricultural Research Center, Giza, 12611 Egypt; 3 The Identification of Microorganisms, Biological Control of Plant Diseases and Evaluation of Biofungicide Unit (IMPCPDEBU), Plant Pathology Research Institute, Agricultural Research Center (ARC), Giza, Egypt; 4 Chemistry of Flavour and Aroma Department, National Research Centre, Cairo, Egypt; 5 Biochemistry Department, Faculty of Agriculture, Beni-Suef University, Beni-Suef, 62511 Egypt

**Keywords:** UV-C irradiation, Apiaceae fruits, Essential oil composition, GC–MS, Antifungal activity

## Abstract

Long-term storage can erode the value of Apiaceae fruits through volatilization and compositional drift of their essential oils (EOs). This study evaluated UV-C pre-treatment (25 min) and packaging type as practical approaches to preserve EO quality in fennel (*Foeniculum vulgare*), anise (*Pimpinella anisum*), cumin (*Cuminum cyminum*), and caraway (*Carum carvi*) fruits stored for 12 months in four packages (jute, polyethylene 120 µm, polyethylene 150 µm, and an antifungal film). EO profiles were monitored by GC–MS at 0, 3, 6, 9, and 12 months; antifungal efficacy was assessed against *Rhizoctonia solani*, *Alternaria solani*, and *Fusarium oxysporum*; and key EO constituents were docked against cutinase (PDB: 1xzb). At baseline, fennel EO was dominated by estragole (49.97% in control; 57.13% after UV-C) and anethole (20.02%); anise EO was rich in anethole (63.79% in control; 64.93% after UV-C); cumin EO was characterized by γ-terpinen-7-al (21.56% in control; 25.89% after UV-C) and cuminaldehyde (19.62% in control; 20.33% after UV-C); and caraway EO contained high levels of carvone (41.60% in control; 47.69% after UV-C) and D-limonene (24.36% in control; 33.11% after UV-C). After 12 months, hermetic packaging (150 µm and antifungal film) better preserved the dominant chemical signature of each EO under UV-C, with estragole at 85.16% in fennel (antifungal), anethole at 72.63% in anise (150 µm), cuminaldehyde at 37.46% in cumin (antifungal), and carvone at 63.14% in caraway (antifungal). Overall, combining UV-C with hermetic/antifungal packaging provides an effective strategy to maintain Apiaceae EO composition and bioactivity during extended storage.

**Supplementary Information:**

The online version contains supplementary material available at 10.1186/s12870-026-09644-x.

## Introduction

One of the most well-known families of flowering plants (Angiosperms) is the Apiaceae, which comprises approximately 300–455 genera and 3000–3750 species distributed worldwide [1]. Asia contains the highest genus richness (≈289 genera), including a large proportion of endemics (≈177), followed by Europe (≈126) and Africa (≈121) (Plunkett et al. 2018). Plants of the Apiaceae family have long been utilized in ethnomedicines [1–3]. Essential oils (EOs) can be extracted from most plant organs; however, in Apiaceae, the highest EO yield is typically associated with the fruits/seeds [4]. EOs are complex mixtures of bioactive compounds, including terpenoids, monoterpenes, sesquiterpenes, aldehydes, ketones, alcohols, phenylpropanoids, and esters. In planta, these metabolites contribute to defense against microbial invasion and oxidative stress, including protection of cellular components from UV-induced damage [5]. Apiaceae fruits’ EOs are widely applied as food flavors, in perfume, dyes, and the manufacture of soaps and detergents, with biological activities that depend strongly on chemical composition [6–12].

Fennel (*Foeniculum vulgare*), anise (*Pimpinella anisum*), cumin (*Cuminum cyminum*), and caraway (*Carum carvi*) are economically important spice plants in the Apiaceae family [13, 14]. Fennel has been used across civilizations for nutritional and medicinal purposes, including relief of digestive disorders and reported antimicrobial and anti-inflammatory effects [15, 16]. The EO yields of fennel fruits typically range from 1.2% to 5.06% [17–19]. These EOs are extensively used as flavoring agents in foods, cosmetics, and pharmaceuticals [20, 21], and commonly contain Estragole (methyl chavicol), trans-anethole, α-phellandrene, and fenchone as major constituents [19, 21]. Anise is historically linked to ancient civilizations, including Egypt, Greece, and Persia [22, 23]. Anise fruits contain up to 6% EO, where anethole may account for up to 90% of the oil, alongside eugenol, anisaldehyde, and anisic acid [23–26]. Furthermore, cumin fruits may reach EO contents up to 7% [27–29], with major constituents such as cumin aldehyde, cymene, and related terpenoids [29–31]. These components underpin cumin’s reported antioxidant and antimicrobial potential, and other biological activities [28, 32–36]. Caraway has been used since antiquity: its use as a digestive aid is mentioned in the Egyptian Ebers Papyrus (Around 1500 BC) and by Dioscorides (AD 40–90) [37]. Caraway fruits produce 5–7.5% volatile oil, composed mainly of carvone (47–81%) and limonene (9–48%), and the oil has reported antifungal, antioxidant, and antibacterial properties with applications across pharmaceutical and cosmetic sectors [38, 39].

Among phytopathogenic fungi, *Rhizoctonia solani*, *Alternaria solani,* and *Fusarium oxysporum* are particularly destructive and are associated with severe crop losses. *R.* *solani i*s a soilborne pathogen causing damping-off and root rot in numerous crops [40, 41]. *A.* *solani* causes early blight, a globally important foliar disease affecting a wide host range [42]. In contrast, *F. oxysporum* typically invades through roots and colonizes vascular tissues, leading to systemic wilt symptoms and substantial yield reductions [43]. Apiaceae EOs (e.g., fennel, anise, cumin, and caraway) have demonstrated antifungal activity in multiple studies [8, 44]. These oils contain monoterpenes, sesquiterpenes, and phenylpropanoids that can inhibit fungal growth through membrane disruption, leakage of cellular contents, and interference with key enzymatic systems [8, 44–48].

Packages and storage conditions are critical determinants of EO yield, chemical composition, and, therefore, biological activity in Apiaceae spices [49–56]. Improper storage can impact spice aroma, flavor, and overall quality [54]. Several technologies have been used to extend the shelf life, including freezing [57], drying [58], radiation [59], and heat treatment [60]. However, temperature- and process-driven changes can alter volatile profiles and compromise sensory integrity [61].

The current standard for bulk spice packaging commonly involves woven polypropylene or jute bags; however, their porosity makes them susceptible to fluctuations in oxygen and humidity during storage [62, 63]. Moisture sensitivity can stimulate microbial activity and accelerate quality degradation [64]. In addition, insects may penetrate jute bags through their loose fibers, promoting infestations and further deterioration [65–68]. Hermetic storage provides low moisture and oxygen transmission rates, thereby limiting oxidative deterioration and suppressing insect development, offering an alternative to gamma irradiation and chemical fumigation [69–71]. Significant reductions in post-harvest losses have been reported for commodities stored in hermetic systems compared with conventional bags, including turmeric, rice, moringa, maize, groundnuts, pulses, cowpea, green coffee, and wheat [72–78].

Commercial fumigation with ethylene oxide can reduce microbial load in spices, yet it is banned in the EU due to toxic and carcinogenic residue concerns [79]. Alternative approaches, such as steam sterilization, may degrade quality, while irradiation technologies (e.g., microwave and gamma) are limited by costs and potential flavor changes [80–83]. UV-C sterilization can inactivate surface contaminants without leaving harmful residues and is increasingly studied for improving hygiene and quality preservation in various foods [83–85]. At low doses, UV-C may act as an abiotic elicitor that stimulates secondary metabolites (e.g., phenolics, flavonoids, carotenoids, ascorbic acid) and antioxidant enzymes, helping mitigate oxidative damage such as membrane lipid peroxidation [49, 54, 86–89]. Mechanistically, UV-C exposure promotes reactive oxygen species (ROS) formation via mitochondrial electron transport and NADPH oxidase, and plants may perceive UV-C through redox shifts; however, excessive ROS may also cause damage if antioxidant defenses are insufficient [90–92].

Despite the commercial relevance of long-term spice storage, limited information is available on the combined effects of UV-C pre-treatment and hermetic packaging on essential oil yield, chemical stability, and antifungal functionality of Apiaceae spices during extended storage. In preliminary work [54], an optimal UV-C exposure time (25–30 min) was identified to enhance the quality of Apiaceae seed stored in standard packaging; however, this study focused on short-term preservation (30 days). To address the need for long-term application, a related investigation by the same authors, Ali et al. [49], directly addresses this critical research gap and evaluates the sustained effects of UV-C pre-treatment on the quality of Apiaceae spices over an extended 12-month storage period in terms of moisture content and gas ratio, microbiology assay, insect infestation, total phenolic content, and antioxidant activity. Building on these findings, the present study is the first to systematically examine the synergistic effects of UV-C pre-treatment combined with different hermetic packaging systems on (i) essential oil yield, (ii) GC–MS-based chemical quality/fingerprinting, and (iii) antifungal activity against major phytopathogens (*R. solani*, *A. solani*, and *F. oxysporum*) after 12 months of storage. In addition, PCA and AHC were used to explore treatment-related patterns, while molecular docking was applied to predict possible interactions between dominant EO constituents and fungal cutinase without implying confirmation of a biological mechanism.

## Materials and methods

### Fruit selection and treatments

Freshly harvested fruits (seeds) of fennel (*Foeniculum vulgare*), anise (*Pimpinella anisum*), cumin (*Cuminum cyminum*), and caraway (*Carum carvi*) were commercially obtained from SEKEM Egypt. These plant materials were the same Apiaceae seed types used in our previous related studies on UV-C treatment and hermetic storage of Apiaceae spices [49, 54]. Based on our previous optimization [54], a UV-C system was used to treat clean, insect-free fruits at 254 nm with an irradiance of 10.5 mW/cm^2^ for 25 min, while non-irradiated fruits (0 min) served as the control.

The samples were not collected from wild populations or protected areas; therefore, no field collection permit, access license, or additional permission for wild plant collection was required. The botanical identity of the received seed lots was confirmed based on morphological seed characteristics by a specialist in medicinal and aromatic plants at the Medicinal and Aromatic Plants Research Department, Horticulture Research Institute, Agricultural Research Center, Giza, Egypt. Because the materials were authenticated commercial seed lots and not wild-collected botanical specimens, voucher specimens were not deposited. All experimental procedures involving plant materials complied with relevant institutional and national guidelines for the use of cultivated/commercial plant materials in research.

**Fig. 1:**
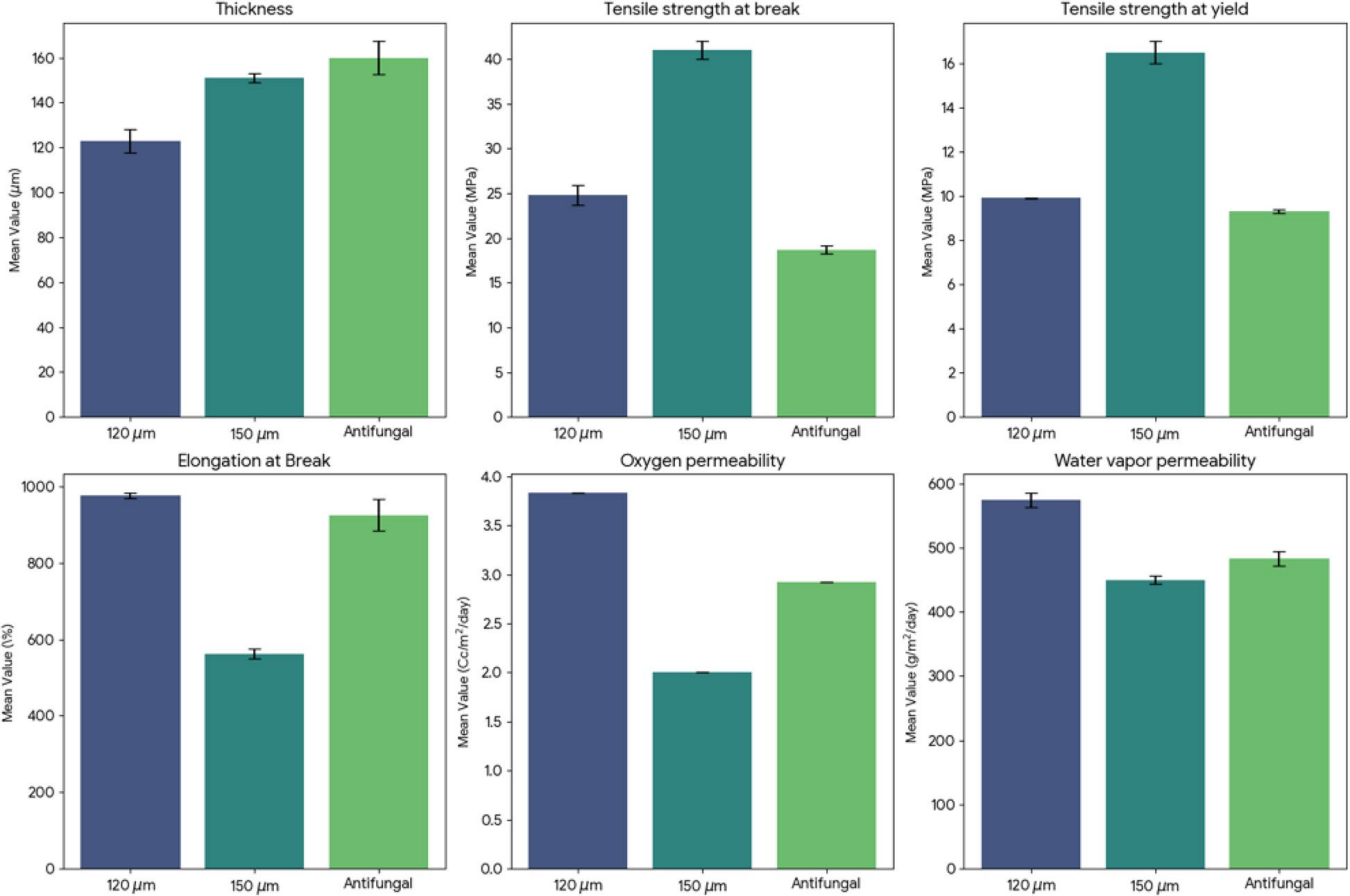
Hermetic bags’ properties [49]. *Values were presented in (Mean ± SD)

All UV-C treatments were applied under the same geometry and distance between lamp and sample, and fruits were spread as a single layer to ensure uniform exposure. Following UV-C treatment, fruits were packaged as described by Ali et al. [49] in multi-layer, hermetically sealed polyethylene bags (Table 1), with 5.0 kg per bag. Three hermetic packages were used: 120 μm, 150 μm, and an antifungal film. The 120 μm and 150 μm hermetic bags were supplied by Shuman Co. (Egypt), while the antifungal hermetic bags were supplied by Sotrafa Co. (Spain). All hermetic bags consisted of a three-layer polyethylene structure; the antifungal bag included an additional antifungal coating layer. The hermetic packages exhibited a very low light transmission (≤ 1 × 10⁻^2^, as specified by the manufacturer). Additional technical characteristics of the hermetic packages are shown in Fig. 1. As a conventional packaging reference, the control treatment was stored in jute bags.

**Table 1:** Fennel essential oil phytochemicals

Components	Irradiate treat	Initial time	3 months				6 months						9 months				12 months			
			Jute	Anti	120 μm	150 μm	Jute	Anti		120 μm		150 μm	Jute	Anti	120 μm	150 μm	Jute	Anti	120 μm	150 μm
Monoterpene hydrocarbons																				
α-Pinene	Control	2.2	0.27	0.59	0.4	0.42	0.89	1.68		0.42		2.12	0	2.54	3.04	2.28	0	1.06	1.92	0.34
	UV-C	2.95	0	0.82	1.12	0.99	1.33	1.16		1.36		2.11	0	1.09	1.3	2.09	0	1.62	0.85	1.51
Sabinene	Control	0.82	0	0.27	0.21	0.2	0.33	0.65		0		0.87	0	0.56	1.03	0.71	0	0	0.7	0
	UV-C	1.12	0	0.4	0.44	0.44	0.43	0.53		0.63		0.76	0	0.67	0.51	0.61	0	0.49	0.4	0.51
β-Pinene	Control	0.41	0	0	0	0	0	0		0		0	0	0	0	0	0	0	0.19	0
	UV-C	0.33	0	0.24	0	0	0	0		0		0	0	0	0.26	0	0	0	0.14	0
β-Myrcene	Control	0.47	0	0	0	0	0	0		0		0.44	0	0.3	0.51	0.33	0	0	0.37	0
	UV-C	0.63	0	0.25	0.24	0.26	0	0		0.33		0.38	0	0.32	0.26	0	0	0	0.21	0.26
α-Phellandrene	Control	0	0	0	0	0	0	0		0		0	0	0	0.68	0	0	0	0	0
	UV-C	0.27	0	0	0	0	0	0		0		0	0	0	0	0	0	0	0	0
p-Cymene	Control	0.84	0.26	0.47	0.47	0.42	0.55	0.77		0.34		0.92	0	0.65	1.07	0.74	0	0.44	0.83	0
	UV-C	0.78	0	0.72	0.55	0.77	0.66	0.78		0.7		0.84	0	0.74	0.21	0.65	0	0.56	0.98	0.65
D-Limonene	Control	11.04	3.03	6.05	4.7	3.44	7.83	9.85		4.81		9.81	0	11.44	13.05	12.54	0	16.36	11.43	2.32
	UV-C	12.82	1.28	8.35	8.01	9.63	9.24	9.6		8.57		11.78	0	12.04	10.73	11.81	0	4.31	4.8	11.12
trans-β-Ocimene	Control	0.72	0	0.26	0.23	0.23	0.3	0.5		0		0.62	0	0.42	0.67	0.44	0	0	0.5	0
	UV-C	0.85	0	0.44	0.41	0.46	0.34	0.51		0.44		0.55	0	0.43	0.44	0.35	0	0.33	0.33	0.37
γ-Terpinene	Control	1.04	0.18	0.33	0.28	0.26	0.37	0.69		0.29		0.81	0	0.57	0.96	0.69	0	0.35	0.75	0
	UV-C	0.97	0	0.61	0.55	0.46	0.4	0.7		0.79		0.73	0	0.69	0.59	0.56	0	0.53	0.45	0.57
γ-Terpinen-7-al	Control	1.41	0	0	0	0	0.52	0		0.33		0.42	0	0	0	0	0	0	0	0
	UV-C	0.68	0	0	0	0	0.34	0		0.77		0	0	0	0	0	0	0	0	0
Oxygenated monoterpenes																				
Eucalyptol	Control	0.99	0.62	0.86	0.93	0.9	0.7	1.14		0.48		1.35	0	1.93	1.38	1.06	0	0.71	1.29	0
	UV-C	1.17	0	0.77	0.75	0.47	0.78	1.07		0.9		1.06	0	0.06	1.1	0.92	0	0.98	0.29	0.97
L-Fenchone	Control	3.69	4.52	4.07	4.84	5.2	4.29	4.56		2.24		4.84	0	3.79	4.93	4.03	0	3.23	4.98	0.65
	UV-C	4.25	1.31	4.02	3.4	4.29	4.91	4.47		3.67		4.01	0	4.23	4.45	3.74	0	4.07	6.44	4.15
Linalool	Control	0	0	0	0	0	0	0		0		0	0	0	0	0	0	0	0	0
	UV-C	0	0	0	0	0	0	0		0		0	0	0	0	0	0	0.65	0	0
cis-Limonene oxide	Control	0	0	0	0.32	0.31	0	0		0		0.34	0	0	0.32	0	0	0	0.3	0
	UV-C	0	0	0	0	0	0	0		0		0	0	0	0.26	0	0	0	0	0
trans-Limonene oxide	Control	0	0	0	0.22	0.23	0	0		0		0	0	0	0.33	0	0	0	0.36	0
	UV-C	0	0	0	0	0	0	0		0		0	0	0	0.25	0	0	0		0
Camphor	Control	0	0.22	0.21	0.3	0.33	0	0		0		0.34	0	0	0.27	0	0	0	0.29	0
	UV-C	0	0	0	0	0	0	0		0		0	0	0	0	0	0	0	0	0
cis-Carveol	Control	0	0	0	0.23	0.26	0	0		0		0.34	0	0	0	0	0	0	0	0
	UV-C	0	0	0	0	0	0	0		0		0	0	0	0	0	0	0	0	0
Cuminaldehyde	Control	0	0.2	0.24	0.19	0.29	1.55	0.62		0.98		1.28	0	0	0	0	0	0	0.89	0
	UV-C	2.14	0	0	0	0	1	0.54		2.51		0.71	0	0	0.37	0	0	0.54	0.87	0.7
(-)-Carvone	Control	1.2	1.34	0.89	1.15	1.14	1.18	0.46		0.37		0.73	0	0	0.35	0	0	0	0.59	0
	UV-C	0.45	0.38	0.59	0.67	0.61	0.95	0.52		0.73		0.5	0	0	0	0	0	0	0.62	0
Anisaldehyde	Control	0.41	2.42	0.94	0.97	0.89	1.84	0.49		0.81		1.01	0	0	0	0	0	0	0	0.8
	UV-C	0	0	0.48	0.49	0.5	1.52	0.49		0.51		0.68	0	0	0	0	0	0	1.05	
Phenylpropanoids																				
Estragole	Control	49.97	44	55.63	50.64	67.37	56.12		75.07		45.96	66.3	0	76.92	69.76	76.36	0	77.84	61.08	77.07
	UV-C	57.13	58.28	69.99	65.32	68.51	57.27		76.35		61.62	67.78	0	79.05	78.5	79.28	0	85.16	73.09	78.29
cis-Anethole	Control	0.64	0.18	0	0	0	0	0		0		0	0	0	0	0	0	0	0	0
	UV-C	0	0	0	0	0	0	0		0		0	0	0	0	0	0	0	0	0
cis-p-Propenylanisole	Control	0	0	0	0	0	0	0		0		0	0	0	0	0	0	0	0	0
	UV-C	0	0	0	0	0	0	0		0		0	0	0	0	0	0	0	0.2	0
Anethole	Control	20.02	36	25.19	30.68	14.9	21.17		3.52	30.74		7.02	0	0.78	1.48	0.82	0	0	7.79	6.59
	UV-C	12.98	35.15	11.27	12.9	11.61	20.44		3.29	13.92		6.92	0	0.75	0.93	0	0	0.76	8.18	0.89
2-Allyl-1,4-dimethoxybenzene	Control	0	0	0	0	0	0	0		0.26		0	0	0	0	0	0	0	0	0
	UV-C	0	0	0	0	0	0	0				0	0	0	0	0	0	0	0	0
Sesquiterpenoids																				
α-Patchoulene	Control	0	0.31	0	0	0	0	0		0		0	0	0	0	0	0	0	0	0
	UV-C	0	0	0	0	0	0	0		0		0	0	0	0	0	0	0	0	0
α-Himachalene	Control	0	0	0	0	0	0	0		0.34		0	0	0	0	0	0	0	0	0.46
	UV-C	0	0	0	0	0	0	0		0		0	0	0	0	0	0	0	0	
Longifolene-(V4)	Control	1.84	2.32	1.48	2.36	0.93	0.72	0		3.78		0	0	0	0	0	0	0	0	6.07
	UV-C	0.09	1.64	0.47	2	0.42	0	0		0.83		0	0	0	0	0	0	0	0.2	0
.β-Copaene.β-Copaene	Control	0	0	0.21	0	0	0	0		0		0	0	0	0	0	0	0	0	0
	UV-C	0	0	0	0	0	0	0		0		0	0	0	0	0	0	0	0.19	0
Zingiberene	Control	0	0.29	0	0.65	0	0	0		0.59		0	0	0	0	0	0	0	0	0.8
	UV-C	0	0	0	0	0	0	0		0		0	0	0	0	0	0	0	0	0
β-Bisabolene	Control	0	0	0	0	0	0	0		0.34		0	0	0	0	0	0	0	0	0.37
	UV-C	0	0	0	0	0	0	0		0		0	0	0	0	0	0	0	0	0
tau.-Cadinol	Control	0	0	0	0	0	0	0		0		0	0	0	0	0	0	0	0.22	0
	UV-C	0	0	0	0	0	0	0		0		0	0	0	0	0	0	0	0	0
Butanoic acid, 2-methyl-, 4-methoxy-2-(3-methyloxiranyl)phenyl ester	Control	0	0	0	0	0	0	0		1.6		0	0	0	0	0	0	0	0	0
	UV-C	0	0	0	0	0	0	0		0		0	0	0	0	0	0	0	0	0
Others																				
trans-Pseudoisoeugenyl 2-methylbutyrate	Control	1.89	3.16	1.96	0.22	1.96	1.63	0		5.32		0.44	0	0	0	0	0	0	5.53	3.77
	UV-C	0.39	1.63	0.57	2.71	0.57	0.39	0		1.73		0	0	0	0	0	0	0	0	0
.2-(1',2'-Epoxypropyl)−4-methoxyphenyl 2-methylbutanoate	Control	0	0	0	0	0	0	0		0		0	0	0	0	0	0	0	0	0.76
	UV-C	0	0	0	0	0	0	0		0		0	0	0	0	0	0	0	0.73	0
3-Hydroxycarbofuran	Control	0.38	0.68	0.33	0	0.33	0	0		0		0	0	0	0	0	0	0	0	0
	UV-C	0	0.33	0	0.45	0	0	0		0		0	0	0	0	0	0	0	0	0
9-Octadecenamide, (Z)-	Control	0	0	0	0	0	0	0		0		0	0	0	0	0	0	0	0	0
	UV-C	0	0	0	0	0	0	0		0		1.18	0	0	0	0	0	0	0	0

Storage began in March 2024. Packaged fruits were stored for 12 months in a chamber under natural ventilation. Essential oil extraction was performed at 0, 3, 6, 9, and 12 months for both irradiated and non-irradiated fruits. During periodic sampling, the probe holes were immediately resealed using specialized plastic sealing tape to maintain hermetic integrity and minimize changes in the internal atmosphere.

### Fruits’ essential oils and GC mass

Essential oil content (%) was determined from stored fruits using hydrodistillation with a Clevenger-type apparatus according to the method recommended in the Egyptian Pharmacopeia [93]. The extraction was conducted at the Laboratory of Medicinal and Aromatic Plants Research Department, Horticulture Research Institute, ARC. Following Guenther [94], 100 g of fruits were ground immediately before extraction and hydrodistilled for 3 h. Essential oil yield was calculated as (volume of oil/mass of dry fruits) × 100, and expressed as % (v/w). Each treatment was analyzed in triplicate, and the reported yield represents the mean value.

For chemical characterization, the extracted oils were analyzed by gas chromatography–mass spectrometry (GC–MS) using an Agilent 8890 GC system coupled to an Agilent 5977B MSD. Oil samples were diluted in hexane at 1:19 (v/v), and 2 μL was injected. Separation was performed on an HP-5MS capillary column (30 m × 0.25 mm i.d., 0.25 μm film thickness). Helium was used as the carrier gas at a constant flow rate (as per instrument setting), with a split ratio of 20:1. The injector temperature was maintained at 280°C. The oven temperature program was set as follows: 50 °C (0 min), increased at 4°C/min to 240 °C (0 min), then increased at 10°C/min to 280 °C and held for 5 min. The MS transfer line (detector interface) temperature was maintained at 280°C.

Mass spectra were acquired by electron ionization (EI) at 70 eV, using a solvent delay of 5 min and a scan range of m/z 40–550. Compound identification was achieved by comparing mass spectral fragmentation patterns with those in the Wiley and NIST mass spectral libraries. Relative composition was expressed as a percentage (%) based on peak area normalization.

### Biological activity

The antifungal activity of the extracted essential oils was evaluated in vitro against three major phytopathogenic fungi: Fusarium oxysporum, Alternaria solani, and Rhizoctonia solani. Essential oils obtained from fruits stored for 0, 6, and 12 months were used for the bioassays. The tested fungal strains were previously isolated and supplied by the Plant Diseases Research Institute, Agricultural Research Center (ARC), Egypt.

To improve oil dispersion, chloroform was added to each essential oil at 2% (v/v) as described by Fahiem [95]. Antifungal activity was assessed using the poisoned food technique. Briefly, each essential oil was added to molten potato dextrose agar (PDA) medium at 45 °C to obtain a final concentration of 0.1% (v/v), then poured into sterile Petri dishes in equal volumes. After solidification, a 5 mm diameter mycelial disc from an actively growing culture of each pathogen was aseptically placed at the center of the plate. Three plates were prepared as replicates for each treatment.

The control consisted of inoculated PDA plates containing chloroform only (without essential oil). All plates were incubated at 25 ± 2 °C for 7–10 days (until the control plates approached full growth). Antifungal activity was expressed as the percentage of mycelial growth inhibition (I%), calculated as:

$$I(\%)=\frac{(C-T)}{C}x 100$$

where I is the percentage inhibition of mycelial growth, C is the mean radial growth (mm) of the fungus in the control plates, and T is the mean radial growth (mm) in the essential-oil-treated plates.

### Molecular docking

A protein–ligand interaction study was conducted to computationally explore possible interactions between major EO constituents and fungal cutinase. The selected ligands included cuminaldehyde, estragole, carvone, γ-terpinen-7-al, and trans-anethole. The crystal structure of cutinase (PDB ID: 1xzb) was retrieved from the Protein Data Bank (RCSB PDB). Before docking, the protein structure was prepared by removing crystallographic water molecules and any co-crystallized ligands, followed by the addition of polar hydrogens and assignment of partial charges to ensure proper interaction calculations.

The three-dimensional structures of the selected ligands were built and geometry-optimized, then saved in PDB format for docking. Ligand rotatable bonds were defined, and the lowest-energy conformations were used as inputs. Molecular docking simulations were performed to predict the preferred binding poses and binding affinities of each compound within the active site of cutinase. All docking runs were executed under identical settings, using default parameters to maintain consistency across ligands. Docking scores and predicted poses were interpreted comparatively and were not considered evidence of actual enzyme binding or inhibition.

### Data analysis

Data were analyzed using IBM SPSS Statistics v26 (IBM Corp., USA). A three-way analysis of variance (ANOVA) was performed to test the effects of radiation treatment (2 levels: UV-C 25 min vs. 0 min), packaging type (4 levels), and storage duration (5 levels: 0, 3, 6, 9, and 12 months), including three independent replicates per treatment combination. Normality and homogeneity of variance were checked before ANOVA, and the level of significance was set at *p ≤* 0.05.

When a significant three-way interaction (radiation × packaging × storage) was detected, the interaction was interpreted by conducting two-way ANOVA (radiation × packaging) separately at each storage time point (0, 3, 6, 9, and 12 months) to clarify how packaging modified the UV-C effect across storage. Where appropriate, mean separation was performed using a post-hoc multiple comparison test at *p ≤* 0.05 (as implemented in SPSS).

Multivariate analyses were conducted using XLSTAT-Pro (Addinsoft), including principal component analysis (PCA) with biplot visualization, and agglomerative hierarchical clustering (AHC) to explore associations and grouping patterns among treatments; these analyses were descriptive and were not used to establish causality [96].

## Results

Due to severe insect infestation and progressive deterioration, the performance of fruits stored in jute bags (both UV-C treated and untreated) could only be assessed up to 6 months. Beyond this point, jute-stored samples were excluded from further analyses because their condition no longer met the minimum quality requirements for reliable EO extraction and compositional evaluation.

### Essential oil percentage

**Fig. 2:**
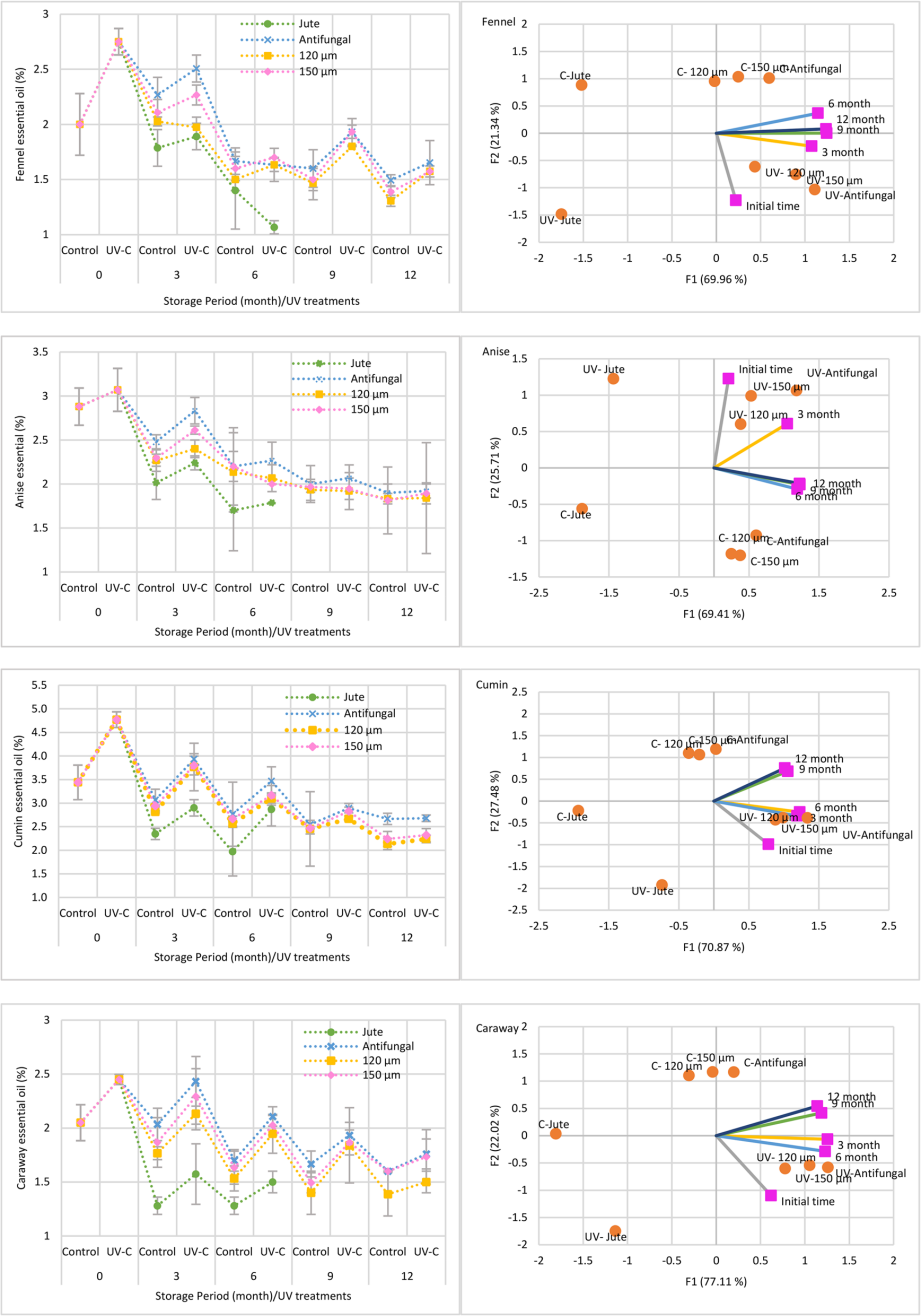
Essential oil content (%) in fennel, anise, cumin, and caraway fruits treated with 0 (C) and 25 min of UV-C and stored in four packaging types (jute, 120 μm, 150 μm, and antifungal) during 12 months. (*P ≤* 0.05)

Changes in essential oil yield (%) of Apiaceae fruits stored under different packaging systems over 12 months are presented in Fig. 2. Overall, essential oil yield declined progressively with storage time, with the largest reduction occurring within the first 3 months across all packaging treatments. Immediately after UV-C exposure (25 min), treated fruits generally showed higher EO yield than untreated fruits, indicating a short-term stimulatory effect or improved extractability.

However, the influence of UV-C on EO retention over time varied by species. In fennel and cumin fruits, UV-C treatment was associated with a faster early decrease in EO yield at 3 and 6 months, suggesting that UV-C may have accelerated the loss of some volatile fractions or promoted early compositional shifts. In contrast, anise and caraway exhibited similar declining trends in treated and untreated samples, indicating that UV-C had a weaker long-term impact on EO retention than packaging type.

Packaging effects were more consistent and pronounced than radiation effects. Fruits stored in jute bags showed the lowest EO yield throughout the monitored period (limited to 0–6 months), followed by those stored in 120 μm hermetic bags. In contrast, fruits packed in 150 μm and antifungal hermetic bags maintained higher EO yield during storage, with the antifungal packaging showing the greatest retention. By month 12, differences among the hermetic packaging treatments became smaller, suggesting that prolonged storage eventually reduced EO yield across treatments, even under improved barrier properties.

Statistical analysis using three-way interaction ANOVA revealed significant differences in essential oil content based on storage time, radiation treatment, and packaging types for fennel and anise fruits (P < 0.05; fennel: F 12,80 = 4.40.2 and anise: F 12,80 = 2.27), while no significant differences were observed for cumin (F 12,80 = 0.597) and caraway (F 12,80 = 1.056). PCA provided an exploratory visualization of treatment-related variation. PCA indicated that the first principal component (F1) explained most of the variability in EO yield patterns for each species: fennel (69.96%), anise (69.41%), cumin (70.87%), and caraway (77.11%). The second component (F2) explained 21.34% (fennel), 25.71% (anise), 27.48% (cumin), and 22.02% (caraway). Treatments using hermetic packaging (120 μm, 150 μm, and antifungal), under both UV conditions (0 and 25 min), loaded positively on F1, whereas jute packaging (0 and 25 min) consistently loaded negatively on F1, reflecting its association with the lowest EO yield over time. The observed loading patterns indicate associations between packaging treatments and EO yield, but do not independently demonstrate a causal preservation effect.

### GC analysis

Tables 1, 2, 3 and 4 show how storage time, packaging type, and UV-C treatment influenced the GC–MS chemical profiles of fennel, anise, cumin, and caraway essential oils. Overall, UV-C–treated fruits generally maintained higher relative proportions of the dominant marker compounds compared with non-irradiated controls. Packaging played the strongest role in preserving EO fingerprints: the antifungal hermetic film consistently provided the best protection, followed by the 150 μm hermetic bag, while jute (evaluated up to 6 months) and 120 μm were less effective, indicating greater susceptibility to volatilization and compositional drift during prolonged storage.

**Table 2:** Anise essential oil phytochemicals

Components	Irradiate treat	Initial time	3 months				6 months				9 months				12 months			
			Jute	Anti	120 μm	150 μm	Jute	Anti	120 μm	150 μm	Jute	Anti	120 μm	150 μm	Jute	Anti	120 μm	150 μm
Monoterpene hydrocarbons																		
p-Cymene	Control	0.25	0	0	0	0	0	0	0	0	0	0	0	0	0	0	0	0
	UV-C	1.16	0	0	0	0	0	0	0	0	0	0	0	0	0	0	0	0
D-Limonene	Control	1.21	0	0	0	0	0	0	0	0	0	0	0	0	0	0.44	0	1.03
	UV-C	1.05	0	0	0.24	0	0	0	0	0	0	0	0.18	0.34	0	0	0	0
γ-Terpinene	Control	0.62	0	0	0	0	0	0	0	0	0	0	0	0	0	0	0	0
	UV-C	2.09	0	0	0	0	0	0	0	0	0	0	0	0	0	0	0	0
γ-Terpinen-7-al	Control	1.79	0	0	0	0	0	0	0	0	0	0	0	0	0	0	0	0
	UV-C	3.35	0	0	0	0	0	0	0	0	0	0	0	0	0	0	0	0
Oxygenated monoterpene																		
Fenchone	Control	0	0	0	0	0	0	0	0	0	0	0	0	0	0	0	0	0.42
	UV-C	0	0	0	0.19	0	0	0	0	0	0	0	0	0	0	0	0	0
Cuminaldehyde	Control	1.66	0	0	0	0	0.76	0.24	0	0.43	0	0.24	0	0	0	0.36	0	0
	UV-C	3.81	0	0	0	0	0.45	0.38	0.62	0.65	0	0	0	0.28	0	0	0	0
(-)-Carvone	Control	4.72	0.26	0.26	0.22	0.31	0	0	0	0	0	0	0	0	0	0	0	0
	UV-C	0.3	0.23	0	0.33	0	0	0	0	0	0	0	0	0	0	0	0	0
Anisaldehyde	Control	0.66	1.34	1.03	1.3	1.47	1.57	0	2.75	2.11	0	3.14	2.69	2.93	0	3.58	3.11	2.02
	UV-C	0.67	1.27	1.23	1.38	1.23	3.94	1.99	2.64	2.08	0	2.53	2.47	3.07	0	2.78	2.54	2.47
Phenylpropanoids																		
Estragole	Control	1.68	4.89	3.16	2.21	3.21	3.69	0.83	1.53	1.65	0	4.55	8.39	2.57	0	10.92	3.08	17.78
	UV-C	1.9	4.53	3.09	1.41	1.92	1.19	1.77	1.59	1.66	0	2.55	6.84	2.71	0	1.84	1.84	1.4
cis-Anethole	Control	0.28	0.33	0.32	0.35	0.4	0.43	1.2	0.35	0.35	0	0.44	0.36	0.42	0	0.27	0.4	0
	UV-C	0.29	0.3	0.34	0.33	0.35	0.29	0	0.41	0.39	0	0.42	0.35	0.44	0	0.34	0.31	0
Anethole	Control	63.79	63.18	68.54	69.96	68.13	64.83	69.35	67.7	68.26	0	65.28	63.86	67.14	0	56.25	65.66	60.38
	UV-C	64.93	67.27	75.53	71.01	71.34	65.37	70.62	67.67	68.23	0	69.69	64.85	69.24	0	69.95	66.82	72.63
2-Allyl-1,4-dimethoxybenzene	Control	0.57	0.56	0.39	0.58	0.66	0.7	0.38	0.68	0.66	0	0.67	0.63	0.65	0	0.64	0.51	0
	UV-C	0.58	0.63	0.39	0.56	0.66	0.64	0.63	0.71	0.66	0	0.66	0.7	0.65	0	0.48	0.57	0
Butanoic acid, 2-methyl-, 4-methoxy-2-(3-methyloxiranyl)phenyl ester	Control	0	0	0	0	0	3.39	1.48	3.23	2.98	0	2.34	2.24	3.61	0	3.36	2.79	1.06
	UV-C	0	0	0	0	0	2.62	1.94	3.26	2.91	0	2.45	2.18	1.42	0	1.89	2.7	1.63
Sesquiterpenoids																		
δ-EIemene	Control	0	0	0	0	0	0	0	0	0	0	0	0	0	0	0	0	0
	UV-C	0	0	0	0	0	0	0	0	0	0	0	0	0.19	0	0	0	
α-Himachalene	Control	0.96	1.1	0.82	1.07	0.84	1.06	1.61	1.02	1	0	1.11	0.95	0.9	0	0.94	0.99	0.76
	UV-C	0.92	1.08	0.96	1	0.98	0.91	0.98	0.96	1.04	0	0.86	1.09	1.09	0	0.98	1.05	0.9
Longifolene-(V4)	Control	8.62	9.88	10.94	10.01	9.21	8.7	9.52	9.17	9.18	0	9.78	8.38	9.48	0	8.72	10.05	8.72
	UV-C	8.5	9.65	7.14	8.69	7.9	8.6	8.63	8.64	9.41	0	8.17	9.73	9.49	0	10.42	10.38	10.16
Aromandendrene	Control	0.46	0.42	0.21	0.52	0.45	0.44	0.35	0.45	0.47	0	0.52	0.44	0.42	0	0.5	0.53	0.45
	UV-C	0.47	0.48	0.48	0.48	0.47	0.45	0.46	0.43	0.47	0	0.4	0.5	0.5	0	0.56	0.56	0.54
Zingiberene	Control	1.81	2.95	1.21	2.04	1.75	1.64	2.36	1.54	1.65	0	1.71	1.34	1.35	0	1.79	2.03	1.64
	UV-C	1.75	1.02	1.78	1.79	1.78	1.73	1.63	1.55	1.66	0	1.32	1.61	1.54	0	2.03	1.97	1.94
β-Himachalene	Control	0.59	0.65	0.74	0.7	0.56	0.62	0.42	0.61	0.66	0	0.72	0.54	0.58	0	0.65	0.72	0.61
	UV-C	0.61	0.67	0.61	0.62	0.62	0.58	0.64	0.61	0.68	0	0.52	0.7	0.67	0	0.73	0.76	0.71
β-Bisabolene	Control	0.86	1.12	0.99	1.08	0.83	1.02	1.7	0.87	0.91	0	0.98	0.84	0.84	0	1.03	1.07	0.81
	UV-C	0.87	1.02	0.91	0.9	0.88	0.96	0.95	0.9	0.95	0	0.84	0.92	0.91	0	1.05	1.05	0.97
γ-Himachalene	Control	0.26	0.36	0	0.35	0.32	0.45	0.27	0.45	0.46	0	0.52	0.42	0.43	0	0.55	0.51	0
	UV-C	0	0.35	0.3	0.31	0.32	0.49	0.41	0.49	0.45	0	0.42	0.47	0.48	0	0	0.48	0
β-Vetivenene	Control	0	0	0	0	0	0.42	0.24	0.52	0.44	0	0.52	0.47	0.43	0	0.51	0	0
	UV-C	0	0	0	0	0	0.42	0.36	0.45	0.42	0	0.46	0.5	0.48	0	0	0	0
Isospathulenol	Control	0	0.31	0	0.24	0.3	0.49	0.24	0.43	0.39	0	0.34	0.34	0.38	0	0.44	0	0
	UV-C	0	0.29	0.26	0.25	0.32	0.49	0.37	0.4	0.38	0	0.4	0.37	0.36	0	0	0.36	0
Other																		
trans-Pseudoisoeugenyl 2-methylbutyrate	Control	6.42	9.48	9.9	8.01	8.31	9.79	9.79	8.7	8.39	0	7.13	8.12	7.86	0	9.04	8.56	4.32
	UV-C	6.32	8.41	6.98	7.87	8.53	10.87	8.25	8.67	7.97	0	8.3	6.53	6.15	0	6.96	8.61	6.64
3-Hydroxycarbofuran	Control	2.79	3.16	1.5	1.36	3.24	0	0	0	0	0	0	0	0	0	0	0	0
	UV-C	0.42	2.8	0	2.64	2.7	0	0	0	0	0	0	0	0	0	0	0	0

**Table 3:** Cumin essential oil phytochemicals

Components	Irradiate treat	Initial time	3 months				6 months				9 months				12 months			
			Jute	Anti	120 μm	150 μm	Jute	Anti	120 μm	150 μm	Jute	Anti	120 μm	150 μm	Jute	Anti	120 μm	150 μm
Monoterpene hydrocarbons																		
β-Thujene	Control	0.34	0.43	0.35	0.33	0.43	0.55	0.44	0	0.3	0	0.56	0.51	0.57	0	0.26	0.24	0.24
	UV-C	0.48	0.38	0.2	0.38	0.33	0.44	0	0.31	0.31	0	0.51	0.57	0.57	0	0	0.31	0
α-Pinene	Control	0.92	1.15	1.01	0.87	1.07	1.45	1.16	0.36	0.82	0	1.42	1.39	1.54	0	0.77	0.72	0.75
	UV-C	1.25	0.97	0.6	1.09	0.91	1.16	0.36	0.85	0.83	0	1.45	1.53	1.55	0	0.61	0.88	0.88
Sabinene	Control	1.07	1.25	0.64	0.95	1.09	1.37	1.37	0.58	1.02	0	1.41	1.41	1.46	0	0.78	0.72	0
	UV-C	1.32	0.86	0.74	1.14	1.09	1.31	0.6	0.99	0.99	0	1.39	1.47	1.49	0	0.57	0	0.89
β-Pinene	Control	9.73	11.1	9.89	9.74	10.92	12.1	12.29	6.72	9.83	0	12.48	12.67	12.68	0	10.64	10.13	10.64
	UV-C	12.63	10.26	8.57	10.8	11.22	11.39	6.82	10.46	9.72	0	13.25	12.82	12.9	0	9.28	11.8	11.08
Ethylhexanol	Control	0	0	0	0	0	0	0	0	0	0	0.55	0	0	0	0	0	0
	UV-C	0	0	0	0	0	0	0	0	0	0	0	0	0	0	0	0	0
β-Myrcene	Control	0.9	0.93	0.82	0.88	0.97	1.06	1.05	0.56	0.78	0	0.96	1.01	1.03	0	0.64	0.59	0.64
	UV-C	1.02	0.8	0.66	0.98	0.78	0.98	0.57	0.78	0.78	0	0.98	1.06	1.07	0	0	0.71	0
α-Phellandrene	Control	0.53	0.48	0.41	0.47	0.46	0.42	0.45	0.32	0.26	0	0.4	0.29	0.37	0	0	0.24	0
	UV-C	0.67	0.41	0.26	0.5	0.4	0.46	0.32	0.38	0.38	0	0.2	0.44	0.33	0	0	0.3	0
α-Terpinene	Control	0.14	0	0	0	0	0	0	0	0	0	0	0	0	0	0	0	0
	UV-C	0	0	0	0	0	0	0	0	0	0	0	0	0	0	0	0	0
p-Cymene	Control	6.81	8.85	8.92	9.55	8.83	10.48	10.92	9.05	11.01	0	10.45	11.99	11.7	0	10.61	11.87	9.92
	UV-C	9.06	9.18	8.39	9.34	8.86	10.16	8.7	9.73	9.4	0	13.56	11.53	12.71	0	9.3	9.3	10.55
D-Limonene	Control	3.06	0.77	0.71	0.82	0.84	0.88	0.93	0.59	0.75	0	1.77	0.93	1.04	0	0.59	0.58	0.91
	UV-C	1.1	0.8	0.75	0.83	0.68	0.85	0.58	0.72	0.75	0	1.09	0.93	0.97	0	0	0.67	0
γ-Terpinene	Control	12.93	13.78	12.48	13.86	13.73	15.52	15.21	12.11	12.1	0	13.88	14.21	14.21	0	12.82	12.01	13.42
	UV-C	17.09	13.88	11.28	13.13	14.01	14.05	11.41	13.65	13.4	0	12.99	15.05	13.72	0	11.65	14.15	11.7
cis-Sabinene hydrate	Control	0	0	0	0	0	0.22	0.23	0.25	0.21	0	0	0.21	0.19	0	0	0	0
	UV-C	0	0	0	0	0	0.16	0.2	0.21	0.22	0	0	0.21	0.2	0	0	0	0
β-Cyclocitral	Control	0	0	0	0	0	0	0	0	0	0	0	0	0	0	0	0	0
	UV-C	0	0	0	0	0	0	0	0	0	0	0	0	0.18	0	0	0	0
Oxygenated monoterpenes																		
Eucalyptol	Control	0.24	0	0.25	0.24	0.27	0.24	0.25	0.28	0.22	0	0.23	0.24	0.23	0	0	0	0
	UV-C	0.23	0.22	0	0.3	0.19	0.22	0.17	0.2	0.21	0	0.22	0.23	0.24	0	0	0	0
Fenchone	Control	0.2	0	0	0	0	0	0	0	0	0	0	0	0	0	0	0	0
	UV-C	0	0	0	0	0	0	0	0	0	0	0	0	0	0	0	0	0
Linalool	Control	0	0.32	0	0	0	0	0	0	0	0	0	0	0	0	0	0	0
	UV-C	0	0	0	0	0	0	0	0	0	0	0	0	0	0	0	0	0
L-Pinocarveol	Control	0	0	0	0	0	0.16	0.15	0.19	0.16	0	0	0	0	0	0	0	0
	UV-C	0	0	0	0	0	0.18	0	0.15	0.15	0	0	0	0	0	0	0	0
E-2-Caren-4-ol	Control	0	0	0	0	0	0.18	0.15	0.19	0.19	0	0	0.21	0.2	0	0	0	0
	UV-C	0	0	0	0	0	0.16	0.16	0.17	0.16	0	0	0	0.21	0	0	0	0
Terpinen-4-ol	Control	0.24	0.35	0.6	0.36	0.34	0.24	0.17	0.27	0.23	0	0.19	0.18	0.2	0	0	0	0
	UV-C	0	0.42	0.42	0.29	0.38	0.21	0.24	0.21	0.24	0	0.2	0.18	0.18	0	0	0	0
3-p-Menthen-7-al	Control	1.36	1.14	0.82	2	1.99	0.7	0.71	0.94	0.9	0	0.69	0.63	0.61	0	0.7	0.66	0.68
	UV-C	0.89	0.91	0.83	2	0.97	0.79	0.95	0.82	0.95	0	0.62	0.66	0.58	0	0.75	0.71	0.72
(-)-Myrtenol	Control	0	0	0	0	0	0.13	0.13	0.17	0	0	0	0	0.22	0	0	0	0
	UV-C	0	0	0	0	0	0.18	0	0	0.15	0	0	0	0	0	0	0	0
3-Isopropylbenzaldehyde	Control	0	0	0	0	0	0	0	0	0	0	0.45	0	0	0	0	0	0
	UV-C	0	0	0	0	0	0	0	0	0	0	0	0	0	0	0	0	0
Cuminaldehyde	Control	19.62	23.47	25.11	24.65	23.8	26.09	25.85	32.36	30.63	0	26.17	28.42	27.73	0	33.17	29.58	32.36
	UV-C	20.33	23.85	26.89	24.55	24.78	28.91	32.1	28.89	29.07	0	29.43	27.76	28.89	0	37.46	31.03	35.11
(-)-Carvone	Control	2.58	0	0	0.43	0.4	0	0	0	0	0	2.53	0	0.65	0	0	0	0
	UV-C	0.52	0	0	0.41	0	0	0	0	0	0	0.89	0	0	0	0	0	0
Phellandral	Control	0	0	0	0	0	0	0	0	0	0	0	0	0	0	0	0	0
	UV-C	0	0	0	0.21	0	0	0	0	0	0	0	0	0	0	0	0	0
α-Terpinen-7-al	Control	9.07	6.92	6.72	13.74	17.67	0	0	0	0	0	0	0	0	0	0	0	0
	UV-C	5.26	6.03	7.25	13.15	5.94	0	0	0	0	0	0	0	0	0	0	0	0
γ-Terpinen-7-al	Control	21.56	25.4	26.46	17.78	14.56	21.81	21.99	26.3	22.76	0	20.44	20.52	19.89	0	23.84	21.49	21.69
	UV-C	25.89	25.36	26.36	17.98	25.36	20.79	26.78	23.84	24.42	0	18.63	19.61	18.93	0	26.06	23.68	23
p-Cymen-7-ol	Control	0	0	0	0	0	0	0	0	0.17	0	0	0	0	0	0	0	0
	UV-C	0	0	0	0	0	0	0.17	0	0	0	0	0	0	0	0	0	0
p-Mentha-1,4-dien-7-ol	Control	0.54	0.73	0.67	0.62	0.36	0.4	0.45	0.62	0.38	0	0.34	0.32	0.35	0	0	0	0
	UV-C	0.56	0.53	0.61	0.56	0.52	0.41	0.66	0.5	0.45	0	0	0.36	0.32	0	0	0	0
Phenylpropanoids																		
cis–p-propenyl anisole	Control	6.48	0.69	2.02	0	0	0	0	0	0	0	0	0	0	0	0	0	0
	UV-C	0	2.91	2.91	0	1.09	0	0	0	0	0	0	0	0	0	0	0	0
Anethole	Control	0	0	0	0	0	4.13	4.15	5.68	4.78	0	3.56	3.36	3.6	0	4.28	10.21	5.99
	UV-C	0	0	0	0	0	4.8	6.75	4.98	5.41	0	3.37	3.74	3.42	0	3.35	4.8	3.89
Sesquiterpenoids																		
β-Gurjunene	Control	0.34	0.45	0.34	0.43	0.39	0	0	0	0	0	0	0	0	0	0	0	0
	UV-C	0.38	0.42	0.46	0.38	0.4	0	0	0	0	0	0	0	0	0	0	0	0
γ-Muurolene	Control	0	0	0	0	0	0.35	0.37	0.38	0.29	0	0.31	0.29	0.28	0	0	0	0.36
	UV-C	0	0	0	0	0	0.37	0.4	0.38	0.31	0	0.2	0.33	0.25	0	0	0.35	0.4
β-Caryophyllene	Control	0.2	0.27	0.27	0.3	0.31	0.21	0.22	0.26	0.25	0	0.19	0.19	0.19	0	0	0	0
	UV-C	0.23	0.26	0.37	0.5	0.24	0.3	0.26	0.25	0.2	0		0.21	0.21	0	0	0	0
(E)-β-Famesene	Control	0.25	0.39	0.38	0.42	0.39	0.3	0.33	0.38	0.48	0	0.3	0.3	0.29	0	0	0	0.38
	UV-C	0.26	0.37	0.64	0.29	0.37	0.49	0.4	0.38	0.32	0	0.33	0.3	0.32	0	0	0	0.39
β-Copaene	Control	0	0	0	0	0	0	0	0	0	0	0	0	0	0	0	0	0
	UV-C	0	0	0	0	0.21	0	0	0	0	0	0	0	0	0	0	0	0
α-Humulene	Control	0	0	0	0	0	0	0	0	0	0	0	0	0	0	0	0	0
	UV-C	0	0	0	0.27	0	0	0	0	0	0	0	0	0	0	0	0	0
4-epi-α-Acoradiene	Control	0.19	0	0	0	0	0.19	0.21	0.24	0.37	0	0	0	0	0	0	0	0
	UV-C	0	0.22	0.27	0	0.2	0.22	0.25	0.25	0.22	0	0	0.18	0	0	0	0	0
Longifolene-(V4)	Control	0	0	0	0	0.59	0	0	0	0	0	0	0	0	0	0	0	0.55
	UV-C	0	0	0	0	0	0	0	0	0	0	0	0	0	0	0	0	0
.(+)-Viridiflorol	Control	0	0	0	0.38	0	0	0	0	0	0	0	0	0	0	0	0	0
	UV-C	0	0	0	0.24	0	0	0	0	0	0	0	0	0	0	0	0	0
Carotol	Control	0.68	1.14	1.13	0.97	0.59	0.82	0.81	1.18	1.12	0	0.72	0.74	0.77	0	0.92	0.97	0.95
	UV-C	0.82	0.96	1.53	0.68	1.07	0.99	1.16	0.9	0.94	0	0.69	0.81	0.74	0	0.96	1.31	0.72
Oxygenated diterpenes																		
Epimanool	Control	0	0	0	0.19	0	0	0	0	0	0	0	0	0	0	0	0	0.51
	UV-C	0	0	0	0	0	0	0	0	0	0	0	0	0	0	0	0	0

**Table 4:** Caraway essential oil phytochemicals

Components	Irradiate treat	Initial time	3 months				6 months				9 months				12 months			
			Jute	Anti	120 μm	150 μm	Jute	Anti	120 μm	150 μm	Jute	Anti	120 μm	150 μm	Jute	Anti	120 μm	150 μm
Monoterpene hydrocarbons																		
α-Pinene	Control	0.27	0	0	0	0	0	0	0	0	0	0	0	0	0	0	0	0
	UV-C	1.36	0	0	0	0.74	0	0	0	0	0	0	0	0	0	0	0	0
β-Pinene	Control	0.34	1.81	0	0.39	0	0	0	0	0	0	0	0	0	0	0	0	0
	UV-C	0	0	0.19	0	0.21	0	0	0	0	0	0	0	0.27	0	0	0	0
β-Myrcene	Control	0.33	0.6	0	0.43	0	0.35	0.44	0.44	0.5	0	0.75	0.6	0.68	0	0	0.36	0.24
	UV-C	0.46	0.41	0.35	0.53	0.23	0.43	0	0.55	0.39	0	0.59	0.49	0.64	0	0	0.35	0.33
p-Cymene	Control	0.45	2.62	0.19	0.84	0	0	0	0	0	0	0	0	0	0	0	0	0
	UV-C	1.12	0.23	0.44	0	0.72	0	0	0	0	0	0	0	0.32	0	0.34	0	0
D-Limonene	Control	24.36	32.89	30.68	32.78	27.81	31.17	32.65	30.79	32.87	0	38.38	35.42	34.65	0	41.92	38.42	30.71
	UV-C	33.11	28.41	21.54	33.33	8.42	27.3	27.23	34.66	29.22	0	34.88	25.84	38.24	0	35.24	34.72	36.02
γ-Terpinene	Control	0.77	2.33	0.24	0.45	0	0	0	0	0	0	0	0.24	0.25	0	0	0	0
	UV-C	1.95	0.24	0.37		0.61	0	0	0	0	0	0	0.33	0.44	0	0	0	0
trans-p-Menth-2,8-dien-1-ol	Control	0	0.47	0.25	0.36	0.24	0	0	0	0	0	0	0	0	0	0	0	0
	UV-C	0.23	0	0.31	0.4	0	0	0	0	0	0	0	0	0	0	0	0	0
E-p-Mentha-2,8-dienol	Control	0	0	0	0	0	0.29	0.35	0.4	0.47	0	0.5	0.29	0.52	0	0	0	0
	UV-C	0	0	0	0	0	0.33	0.41	0.37	0.47	0	0.58	0	0.49	0	0.08	0	0.27
trans-2,8-p-Mentha-dien-1-ol	Control	0	0	0	0	0	0	0.29	0.31	0.37	0	0.35	0.22	0.35	0	0	0	0
	UV-C	0	0	0	0	0	0.27	0.33	0	0.37	0	0.34	0.22	0.32	0	0	0	0
cis-p-Mentha-2,8-dien-1-ol	Control	0	0.34	0.21	0.28	0.21	0	0	0	0	0	0	0	0	0	0	0	0
	UV-C	0	0	0.26	0.31	0	0	0	0	0	0	0	0	0	0	0	0	0
Oxygenated monoterpenes																		
Fenchone	Control	0.71	0	0	0	0	0.25	0	0	0	0	0	0	0	0	0	0	0
	UV-C	0	0	0	0	0	0	0	0	0	0	0	0	0	0	0	0	0
cis-Limonene oxide	Control	0.25	0.32	0	0.36	0	0.29	0.36	0.36	0.42	0	0.44	0.34	0.45	0	0	0.27	0.24
	UV-C	0.25	0.18	0.29	0.33	0	0.32	0.33	0.39	0.45	0	0.47	0.22	0.42	0	0	0.32	0.3
(E)-Limonene oxide	Control	0	0	0	0	0	0.3	0.38	0.37	0.45	0	0.49	0.36	0.5	0	0	0.32	0.33
	UV-C	0	0	0	0	0	0.34	0.37	0.35	0.46	0	0.51	0	0.5	0	0	0.41	0.34
trans-Limonene oxide	Control	0.27	0.36	0.19	0.38	0.23	0	0	0	0	0	0	0	0	0	0	0	0
	UV-C	0.28	0.25	0.32	0.38	0	0	0	0	0	0	0	0	0	0	0	0	0
p-Mentha-1,8-dien-7-ol	Control	0	0.32	0	0.3	0	0	0	0	0	0	0	0	0	0	0	0	0
	UV-C	0	0	0	0	0	0	0	0	0	0	0	0	0	0	0	0	0
trans-Dihydrocarvone	Control	0.27	0.48	0	0.38	0.22	0.3	0.39	0.36	0.43	0	0.42	0.32	0.4	0	0	0	0.24
	UV-C	0.25	0.24	0.35	0.44	0	0.34	0.41	0.4	0.5	0	0.42	0	0.36	0	0	0.36	0.32
trans-Carveol	Control	0.52	0.84	0.41	0.73	0.48	0.47	0.68	0.6	0.87	0	0.63	0.51	0.61	0	0	0.49	0
	UV-C	0.42	0.41	0.67	0.73	0	0.64	0.88	0.74	0.81	0	0.69	0	0.65	0	0.1	0	0.52
Dihydrocarveol	Control	0	0.42	0.21	0.36	0.26	0	0.3	0.28	0.41	0	0.4	0	0.42	0	0	0	0
	UV-C	0	0	0.34	0.4	0	0.33	0.37	0.33	0.46	0	0.39	0	0	0	0	0	0
cis-Carveol	Control	0	0	0	0	0	0.56	0.32	0.29	0.39	0	0.32	1.33	0.34	0	0	0	0
	UV-C	0	0	0	0	0	0.32	0.41	0.33	0.44	0	0.35	1.3	0	0	0	0	0
Carveol	Control	0	0.34	0.2	0.34	0	0	0	0	0	0	0	0	0	0	0	0	0
	UV-C	0	0	0.31	0.33	0	0	0	0	0	0	0	0	0	0	0	0	0
Cuminaldehyde	Control	1.86	0	3.24	3.39	0.81	0	0	0	0	0	0	0	0	0	2.46	1.39	14.67
	UV-C	6.31	2.68	0	1.17	0	0	0	0	0	0	0	0	0	0	0.74	1.29	1.23
Carvone	Control	41.6	49.09	57.78	54.64	60.09	48.65	56.34	54.73	57.93	0	54.31	47.6	51.99	0	54.94	58.28	44.02
	UV-C	47.69	56.72	72.76	58.98	70.09	52.71	63.73	57.31	61.28	0	55.88	53.84	51.91	0	63.14	61.19	59.95
p-Mentha-1,8-dien-7-al	Control	0.3	0.49	0.2	0.42	0.28	0	0	0	0	0	0	0	0	0	0	0	0
	UV-C	0.32	0.27	0.42	0.48	0	0	0	0	0	0	0	0	0	0	0	0	0
L-Perillaldehyde	Control	0	0	0	0	0	0.33	0.42	0.43	0.48	0	0	0	0	0	0	0	0.25
	UV-C	0	0	0	0	0	0.38	0.5	0.44	0.56	0	0	0	0	0	0	0.35	0.32
γ-Terpinen-7-al	Control	1.65	0.94	1.81	1.54	0.29	0	0	0.3	0	0	0	0.96	0.68	0	0.68	0.47	6.67
	UV-C	4.17	6.03	0.37	0.56	0	3.34	0	0	0	0	0	0.97	0.53	0	0.27	0.38	0.39
Phenylpropanoids																		
Estragole	Control	19.31	1.57	1.15	1.11	0.65	0	0	0	0	0	0	0	0	0	0	0	0
	UV-C	0.76	1.46	0.93	1.27	0	0	0	0	0	0	0	0	0	0	0	0	0
cis-Anethole	Control	0	0	0	0	0	14.47	5.14	7.38	3.26	0	1.06	1.83	1.92	0	0	0	0.81
	UV-C	0	0	0	0	0	9.07	3.79	3.14	3.18	0	1.53	1.22	1.59	0	0.09	0.63	0
Anethole	Control	6.77	3.81	0.95	0.84	0.98	2.59	1.93	2.96	1.15	0	1.25	8.54	5.68	0	0	0	1.81
	UV-C	1.33	2.44	0.7	1.23	11.14	3.86	1.24	0.96	1.4	0	2.43	6.15	2.51	0	0	0	0
(E)−4-Methoxy-2-(prop-1-en-1-yl)phenyl 2-methylbutanoate	Control	0	0	0	0	0	0	0	0	0	0	0	0.46	0	0	0	0	0
	UV-C	0	0	0	0	0	0	0	0	0	0	0	3.14	0	0	0	0	0
Sesquiterpenoids																		
α-Himachalene	Control	0	0	0	0	0	0	0	0	0	0	0	0	0	0	0	0	0
	UV-C	0	0	0	0	0	0	0	0	0	0	0	0.31	0	0	0	0	0
Longifolene-(V4)	Control	0	0	0	0	0	0	0	0	0	0	0	0.46	0	0	0	0	0
	UV-C	0	0	0	0	0	0	0	0	0	0	0	3.14	0	0	0	0	0
Zingiberene	Control	0	0	0	0	0	0	0	0	0	0	0	0	0	0	0	0	0
	UV-C	0	0	0	0	0	0	0	0	0	0	0	0.46	0	0	0	0	0
β-Bisabolene	Control	0	0	0	0	0	0	0	0	0	0	0	0	0	0	0	0	0
	UV-C	0	0	0	0	0	0	0	0	0	0	0	0.24	0	0	0	0	0
Other																		
Butanoic acid, 2-methyl-, 4-methoxy-2-(3-methyloxiranyl)phenyl ester	Control	0	0	0	0	0	0	0	0	0	0	0	0	0	0	0	0	0
	UV-C	0	0	0	0	0	0	0	0	0	0	0	0.89	0	0	0	0	0

Across species, GC–MS identified 29 compounds in fennel, 25 in anise, 42 in cumin, and 34 in caraway. Fennel EO was dominated by estragole, which showed a clear increase toward 12 months, likely reflecting a relative enrichment as minor volatiles declined. Anise EO remained strongly characterized by trans-anethole, with the highest stability at 6–9 months, particularly under antifungal and 150 μm packaging. In cumin EO, cuminaldehyde reached its maximum proportion at 9 months, while caraway EO was governed by carvone and D-limonene: limonene followed a variable but slightly increasing trend toward 12 months, whereas carvone rose sharply by 3 months and then remained consistently high, supporting the idea that long-term storage mainly shifts relative composition while hermetic barriers and UV-C help retain the defining chemical signature.

### Hierarchical cluster analysis

**Fig. 3:**
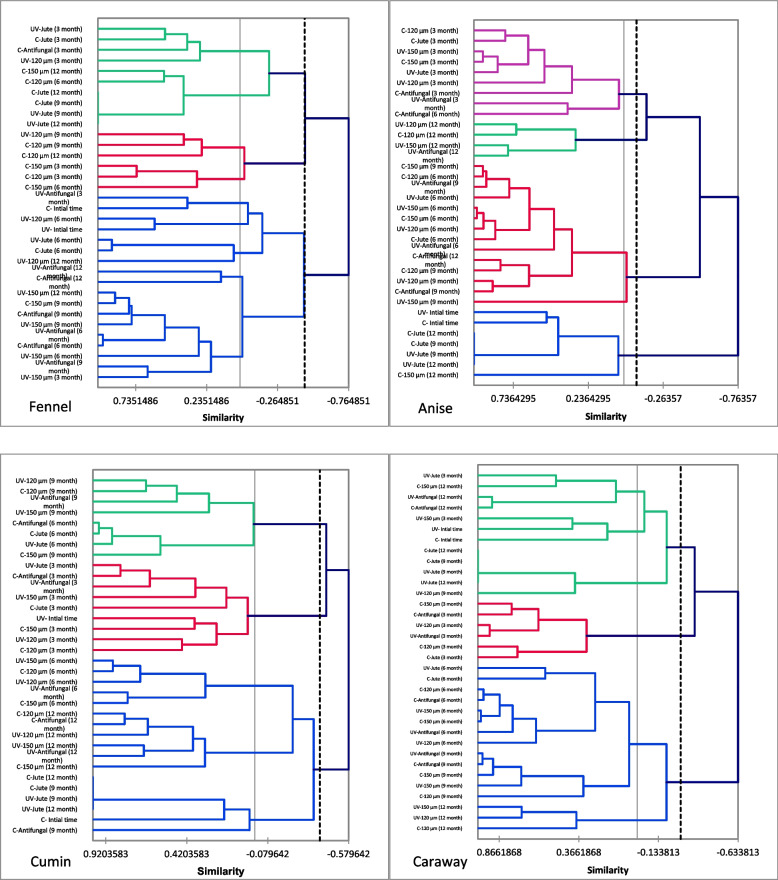
The Hierarchical Cluster dendrogram in fennel, anise, cumin, and caraway fruits treated with 0 (C) and 25 min of UV-C and stored in four packaging types (jute, 120 μm, 150 μm, and antifungal) during 12 months (*P ≤* 0.05)

Hierarchical cluster analysis (HCA) dendrograms (Fig. 3A–D) revealed a highly consistent clustering structure across fennel, anise, cumin, and caraway, where storage duration represented the main source of sample separation captured by the analysis. In all species, the most distinct clusters were formed according to time, and the initial-time samples (0 month; control or UV-C) were frequently positioned far from the other groups at high dissimilarity—particularly in anise and caraway—a pattern consistent with marked changes in the overall quality and chemical profiles during storage. Samples stored for 3 and 6 months tended to cluster together, notably in fennel and cumin, and joined the 9–12-month clusters only at higher dissimilarity levels, indicating that late-storage samples exhibited different multivariate profiles from those recorded during early-to-mid storage.

Within these time-associated clusters, UV-C and non-irradiated treatments at the same storage point were often positioned close to each other, suggesting a stronger association of the clustering pattern with storage duration than with UV-C treatment. For instance, in fennel, the 12-month UV-C and control samples clustered together before merging with the 9-month group. Packaging-associated separation was generally less pronounced, as samples stored in different hermetic packages at the same time point were frequently grouped closely, such as cumin samples stored in 120 and 150 μm bags at 12 months. Nevertheless, some packaging-related patterns were observed: antifungal-packaged samples of anise and caraway showed noticeable separation but remained associated with the broader 12-month grouping. Overall, the HCA patterns suggest that storage duration accounted for a substantial proportion of the variation represented in the dataset, while UV-C treatment and packaging were associated with additional within-time differences. These exploratory groupings describe similarities among samples but do not establish causal relationships among the studied factors and quality changes.

### Antifungal efficacy of essential oil

**Fig. 4:**
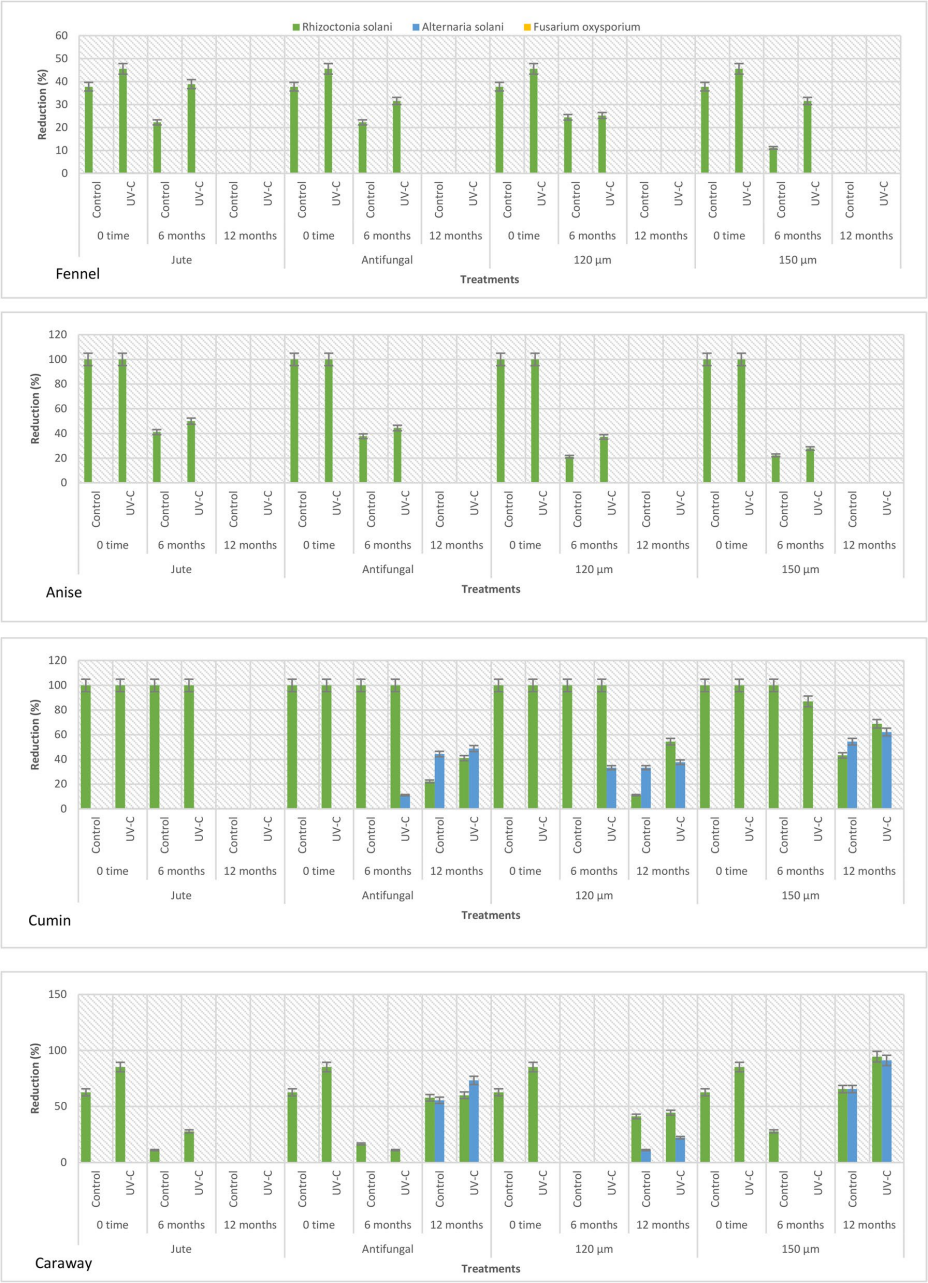
Antifungal activity of essential oil of fennel, anise, cumin, and caraway fruits treated with 0 (control) and 25 min of UV-C and stored in four packaging types (jute, 120 μm, 150 μm, and antifungal) during 12 months (*P ≤* 0.05)

Figure 4 illustrates the antifungal efficacy of essential oils extracted from Apiaceae fruits subjected to UV-C treatment and stored for up to 12 months under different packaging systems. Overall, among the three tested pathogens, *Fusarium oxysporum* was consistently the most resistant to all oils. In general, UV-C pre-treatment (25 min) enhanced antifungal performance, particularly against *Rhizoctonia solani* and *Alternaria solani*. Fennel and anise oils showed strong suppression of *R. solani* across all packaging types up to 6 months; however, their efficacy declined markedly with prolonged storage, and by 12 months, the oils largely failed to inhibit fungal growth. In contrast, cumin and caraway oils maintained higher inhibitory activity for longer periods, achieving sustained reductions against *R. solani* and *A. solani* up to 12 months, especially under improved barrier packaging. Notably, jute-stored UV-C samples exhibited a sharp reduction in antifungal performance within the first 6 months, confirming the poor protective capacity of porous packaging. Among hermetic systems, 150 μm films provided the most effective long-term preservation of antifungal activity for cumin and caraway oils, making them the most suitable option for extended storage control of both *R. solani* and *A. solani.*

### Docking evaluation against cutinase (1xzb)

**Fig. 5:**
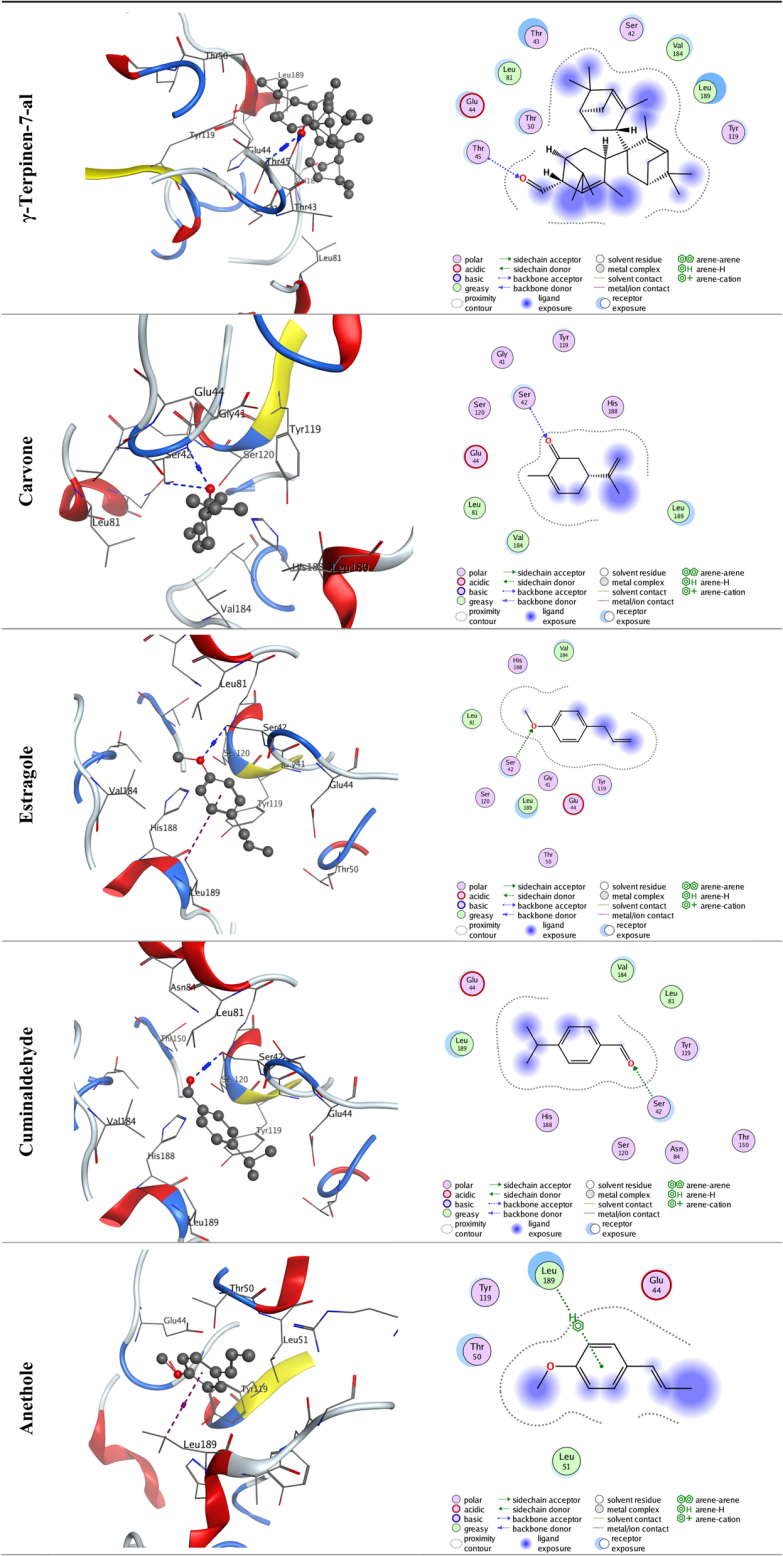
2D & 3D interaction of γ-Terpinen-7-al, Carvone, Estragole, Cuminaldehyde, and Anethole against Cutinase (1xzb) active spots

Docking evaluation against cutinase (1xzb). Table 5 summarizes the ligand–protein interface parameters, including binding energies, interacting residues, and bond lengths. The predicted binding energies for γ-terpinen-7-al, carvone, estragole, cuminaldehyde, and anethole docked into cutinase (PDB: 1xzb) were − 4.8528, − 4.5315, − 4.4446, − 4.4339, and − 4.2273 kcal/mol, respectively, where more negative values indicate stronger predicted interactions. Accordingly, the interaction strength followed the order γ-terpinen-7-al > carvone > estragole > cuminaldehyde > anethole, which showed a qualitative correspondence with the in vitro antifungal trends but did not establish a causal relationship. In contrast, D-limonene did not establish stable contacts within the binding site and showed a negligible docking score, indicating limited predicted affinity under the applied computational conditions. Figure 5 illustrates the binding poses and the major residue contacts stabilizing each ligand within the cutinase active-site region. These docking results are exploratory and do not confirm actual cutinase binding or inhibition.

**Table 5:** Docking interaction data calculations of compounds (γ-Terpinen-7-al, Carvone, Cis-p-Propenylanisole, Estragole, Cuminaldehyde, and Anethole) inside Cutinase (1xzb) active spots

Molecules	Ligand	Receptor	Interaction	Distance (in Å from main residue)	E (Kcal/mol)	S (Kcal/mol)
γ-Terpinen-7-al	O15	THR 45	H-acceptor	2.83	−2.1	−4.8528
	O15	THR 45	H-acceptor	2.76	−2.1	
Carvone	O7	SER 42	H-acceptor	2.72	−1.4	−4.5315
Estragole	O19	SER 42	H-acceptor	2.98	−0.8	−4.4446
Cuminaldehyde	O13	SER 42	H-acceptor	2.78	−1.7	−4.4339
Anethole	6-ring	LEU 189	pi-H	4.38	−0.7	−4.2273

## Discussion

This study provides a comprehensive evaluation of how UV-C pre-treatment combined with hermetic storage influences the chemical stability, temporal evolution, and antifungal functionality of essential oils (EOs) derived from fennel, anise, cumin, and caraway fruits. Across 12 months, EO yield declined in all species, yet UV-C-treated fruits generally maintained higher EO yield and higher relative abundance of key marker compounds than untreated fruits (Fig. 2, Tables 1, 2, 3 and 4). These outcomes support the view that Apiaceae EOs behave as dynamic chemical matrices associated with pre-storage abiotic elicitation (UV-C), package permeability, internal oxygen availability, and species-specific phytochemical structures. This integrated perspective is consistent with the growing consensus that EO profiles respond collectively to storage-driven volatility and oxidation, rather than changing in a purely linear manner [54, 87, 92, 97]. Although studies investigating UV-C effects on medicinal seed EOs remain limited, UV-C is recognized as a non-thermal, residue-free technology that can preserve quality without causing major physical deterioration, consistent with findings in cumin and black pepper volatiles [98, 99]. Immediately after irradiation, all tested species showed a transient increase in EO yield, which may be consistent with a UV-C hormetic response involving oxidative signaling and stimulation of phenylpropanoid and terpenoid pathways [100–102]. However, as storage progressed, EO yield decreased across all species, with the largest decline occurring within the first three months, which agrees with the known instability of monoterpene hydrocarbons that readily undergo evaporation, oxidative cleavage, and polymerization because of their low molecular mass and unsaturation [103]. Similar decreases in essential oil content and shifts in key ratios (e.g., limonene/carvone) during storage have been reported in caraway and other aromatic seeds under different packaging systems [104–107].

The present findings indicate that hermetic storage was more effective than conventional jute packaging for maintaining the quality of Apiaceae fruits during long-term storage. Hermetic packaging reduced EO losses and helped preserve chemical integrity over 12 months, which may be associated with reduced oxygen ingress and moisture exchange and consequent limitation of autoxidation, moisture-related reactions, and photodegradation [69]. In contrast, porous jute bags were associated with greater gas exchange, light exposure, volatilization, and oxidative deterioration. PCA and AHC separated several jute-stored samples from hermetically stored samples, providing an exploratory visualization of treatment-related differences rather than confirming the mechanisms responsible for preservation. Among hermetic systems, antifungal film followed by the 150 μm bag showed the best preservation of EO yield and compositional stability, which may reflect lower moisture migration, more stable internal temperature and relative humidity, improved headspace gas ratios, and a thicker barrier structure [49, 108]. Species-specific behavior also followed predictable chemical patterns. In fennel, estragole progressively enriched during storage in hermetic bags, consistent with the relative stability of aromatic ethers compared with monoterpene hydrocarbons; UV-C further enhanced estragole retention, which may be associated with changes in phenylpropanoid metabolism [88]. In anise, the high stability of anethole reflects the resilience of its methoxy-phenylpropene structure, and hermetic packaging minimized oxidative conversion to anisaldehyde that was more evident in high-oxygen storage [24]. Cumin exhibited the greatest chemical dynamism, where cuminaldehyde increased during mid-storage, possibly reflecting transformation from related precursor compounds, such as γ-terpinen-7-al, under conditions influenced by package permeability [27, 109]. Caraway showed strong compositional resilience; the early increase in carvone may reflect oxidative transformation of limonene during maturation-like storage processes, while hermetic conditions were associated with a more moderate compositional shift [38, 39].

The antifungal results generally corresponded with the chemical profiles retained during storage. Oils obtained from fruits stored in hermetic systems exhibited stronger inhibition of *Alternaria solani* and *Rhizoctonia solani* and retained higher proportions of oxygenated monoterpenes and phenylpropanoids, including carvone, cuminaldehyde, estragole, and anethole, which have been reported to affect fungal membranes, oxidative balance, and ergosterol biosynthesis [45, 110]. The improved antifungal activity of UV-C-treated fruits may be associated with the initial enrichment of oxygenated terpenoids following irradiation. Essential oils and their constituents have been reported to disrupt fungal cell walls and membranes, increase permeability, damage organelles, and cause leakage of intracellular macromolecules, thereby inhibiting spore germination and mycelial growth [111]. Additional proposed mechanisms include lipophilic partitioning into membranes and suppression of genes involved in energy metabolism and mitochondrial function [112, 113]; however, these mechanisms were not directly examined in the present study. Docking analysis provided exploratory predictions of possible interactions between γ-terpinen-7-al, carvone, estragole, cuminaldehyde, anethole, and the cutinase binding region. These predicted affinities do not confirm actual ligand binding, cutinase inhibition, or a causal role in the observed antifungal activity. Similarly, the negligible predicted interaction of limonene should not be interpreted as evidence of a limited contribution to the activity of the complete essential oil mixture [114].

## Conclusion

This study shows that the long-term stability and antifungal effectiveness of essential oils from fennel, anise, cumin, and caraway are driven primarily by storage time and packaging barrier properties, while UV-C acts as a supportive pre-treatment that improves retention of key constituents. Although essential oil yield declined in all species over 12 months—most sharply within the first three months—UV-C-treated fruits generally exhibited higher initial yields and better preservation of dominant marker compounds than untreated fruits. Hermetic packaging clearly outperformed jute storage by limiting oxygen and moisture exchange and thereby reducing volatilization and oxidative degradation; among the tested systems, the antifungal film followed by the 150 μm hermetic bag provided the best preservation of both oil yield and GC–MS chemical fingerprinting. These chemical advantages translated into stronger antifungal activity, particularly against *Rhizoctonia solani* and *Alternaria solani* (with *Fusarium oxysporum* being the most resistant), and docking analysis supported a mechanistic basis by showing stronger predicted binding of oxygenated monoterpenes and phenylpropanoids (γ-terpinen-7-al, carvone, estragole, cuminaldehyde, anethole) to cutinase compared with limonene. Overall, UV-C pre-treatment combined with antifungal or 150 μm hermetic packaging was associated with improved retention of EO yield, chemical profiles, and in vitro antifungal activity during storage. PCA and AHC illustrated treatment-related associations, while molecular docking identified possible ligand–cutinase interactions. These exploratory analyses do not establish causal preservation mechanisms or confirm cutinase inhibition; further biochemical and biological validation is required.

## Supplementary Information

Supplementary Material 1.

## Data Availability

The datasets generated and/or analyzed during the current study are available from the corresponding author on reasonable request.
