# Supplementary material for: UV-C irradiation and hermetic storage modulate essential oil profiles and antifungal bioactivity of Apiaceae fruits during long-term storage
Source: BMC Plant Biol. 2026 Aug 15;26:1394. doi: 10.1186/s12870-026-09644-x (PMC13476923; doi:10.1186/s12870-026-09644-x)
Supplement: Supplementary file 1 [file 12870_2026_9644_MOESM1_ESM.pdf]

```
UNIANOVA Fe BY ST RD PT
  /METHOD=SSTYPE(3)
  /INTERCEPT=INCLUDE
  /POSTHOC=ST RD PT(TUKEY DUNCAN)
  /EMMEANS=TABLES(OVERALL)
  /EMMEANS=TABLES(ST) COMPARE ADJ(LSD)
  /EMMEANS=TABLES(RD) COMPARE ADJ(LSD)
  /EMMEANS=TABLES(PT) COMPARE ADJ(LSD)
  /EMMEANS=TABLES(ST*RD)
  /EMMEANS=TABLES(ST*PT)
  /EMMEANS=TABLES(RD*PT)
  /EMMEANS=TABLES(ST*RD*PT)
  /PRINT=HOMOGENEITY DESCRIPTIVE
  /CRITERIA=ALPHA(.05)

  /DESIGN=ST RD PT ST*RD ST*PT RD*PT ST*RD*PT.
```

Univariate Analysis of Variance

Notes

Output Created		29-Nov-2025 08:30:24
Comments		
Input	Active Dataset	DataSet0
	Filter	<none>
	Weight	<none>
	Split File	<none>
	N of Rows in Working Data File	120
Missing Value Handling	Definition of Missing	User-defined missing values are treated as missing.
	Cases Used	Statistics are based on all cases with valid data for all variables in the model.

Syntax		UNIANOVA Fe BY ST RD PT<br>/METHOD=SSTYPE(3)<br>/INTERCEPT=INCLUDE<br>/POSTHOC=ST RD PT(TUKEY DUNCAN)<br>/EMMEANS=TABLES(OVERALL)<br>/EMMEANS=TABLES(ST) COMPARE ADJ(LSD)<br>/EMMEANS=TABLES(RD) COMPARE ADJ(LSD)<br>/EMMEANS=TABLES(PT) COMPARE ADJ(LSD)<br>/EMMEANS=TABLES(ST*RD)<br>/EMMEANS=TABLES(ST*PT)<br>/EMMEANS=TABLES(RD*PT)<br>/EMMEANS=TABLES(ST*RD*PT)<br>/PRINT=HOMOGENEITY DESCRIPTIVE<br>/CRITERIA=ALPHA(.05)<br>/DESIGN=ST RD PT ST*RD ST*PT RD*PT ST*RD*PT.
Resources	Processor Time	00:00:00.125
	Elapsed Time	00:00:00.094

[DataSet0]

Warnings

Post hoc tests are not performed for Radiationdose because there are fewer than three groups.

Between-Subjects Factors

		N
StorageTime	0 time	24
	12 mon	24
	3 mont	24
	6 mont	24
	9 mont	24

Radiationdose	0 UV	60
	UV	60
PackagingType	120	30
	150	30
	Anti	30
	Jute	30

Descriptive Statistics

Dependent Variable:Fennel

StorageTime	Radiationdose	PackagingType	Mean	Std. Deviation	N
0 time	0 UV	120	2.0000	.28844	3
		150	2.0000	.28844	3
		Anti	2.0000	.28844	3
		Jute	2.0000	.28844	3
		Total	2.0000	.24599	12
	UV	120	2.7467	.12220	3
		150	2.7467	.12220	3
		Anti	2.7467	.12220	3
		Jute	2.7467	.12220	3
		Total	2.7467	.10421	12
	Total	120	2.3733	.45443	6
		150	2.3733	.45443	6
		Anti	2.3733	.45443	6
		Jute	2.3733	.45443	6
		Total	2.3733	.42376	24
12 mon	0 UV	120	1.6533	.20133	3
		150	1.4933	.04619	3
		Anti	1.3067	.04619	3
		Jute	.0000	.00000	3
		Total	1.1133	.68942	12
	UV	120	1.5733	.04619	3
		150	1.5733	.04619	3
		Anti	1.3867	.04619	3

		Jute	.0000	.00000	3
		Total	1.1333	.68889	12
	Total	120	1.6133	.13779	6
		150	1.5333	.06022	6
		Anti	1.3467	.06022	6
		Jute	.0000	.00000	6
		Total	1.1233	.67408	24
3 mont	0 UV	120	2.0267	.04619	3
		150	2.2667	.09238	3
		Anti	2.1067	.12220	3
		Jute	1.8933	.04619	3
		Total	2.0733	.15802	12
	UV	120	1.9733	.04619	3
		150	2.2667	.09238	3
		Anti	1.7867	.16653	3
		Jute	2.5067	.12220	3
		Total	2.1333	.30383	12
	Total	120	2.0000	.05060	6
		150	2.2667	.08262	6
		Anti	1.9467	.21860	6
		Jute	2.2000	.34595	6
		Total	2.1033	.23881	24
6 mont	0 UV	120	1.6000	.10000	3
		150	1.6667	.11547	3
		Anti	1.5000	.20000	3
		Jute	1.4000	.34641	3
		Total	1.5417	.21088	12
	UV	120	1.6333	.05774	3
		150	1.7000	.00000	3
		Anti	1.6333	.15275	3
		Jute	1.0667	.05774	3
		Total	1.5083	.27784	12
	Total	120	1.6167	.07528	6
		150	1.6833	.07528	6
		Anti	1.5667	.17512	6

		Jute	1.2333	.28752	6
		Total	1.5250	.24182	24
9 mont	0 UV	120	1.9333	.11547	3
		150	1.5000	.10000	3
		Anti	1.4667	.15275	3
		Jute	.0000	.00000	3
		Total	1.2250	.76885	12
	UV	120	1.8000	.00000	3
		150	1.9333	.05774	3
		Anti	1.6000	.17321	3
		Jute	.0000	.00000	3
		Total	1.3333	.81724	12
	Total	120	1.8667	.10328	6
		150	1.7167	.24833	6
		Anti	1.5333	.16330	6
		Jute	.0000	.00000	6
		Total	1.2792	.77795	24
Total	0 UV	120	1.8427	.23651	15
		150	1.7853	.33911	15
		Anti	1.6760	.36176	15
		Jute	1.0587	.93477	15
		Total	1.5907	.61526	60
	UV	120	1.9453	.44275	15
		150	2.0440	.44273	15
		Anti	1.8307	.50618	15
		Jute	1.2640	1.22466	15
		Total	1.7710	.77630	60
	Total	120	1.8940	.35265	30
		150	1.9147	.40920	30
		Anti	1.7533	.43938	30
		Jute	1.1613	1.07553	30
		Total	1.6808	.70332	120

Levene's Test of Equality of Error Variances[a]

Dependent Variable:Fennel

F	df1	df2	Sig.
3.732	39	80	.000

Tests the null hypothesis that the error variance of the dependent variable is equal across groups.

a. Design: Intercept + ST + RD + PT + ST * RD + ST * PT + RD * PT + ST * RD * PT

Tests of Between-Subjects Effects

Dependent Variable:Fennel

Source	Type III Sum of Squares	df	Mean Square	F	Sig.
Corrected Model	57.229[a]	39	1.467	71.744	.000
Intercept	339.024	1	339.024	1.658E4	.000
ST	27.708	4	6.927	338.670	.000
RD	.976	1	.976	47.699	.000
PT	11.258	3	3.753	183.469	.000
ST * RD	2.471	4	.618	30.197	.000
ST * PT	13.636	12	1.136	55.557	.000
RD * PT	.101	3	.034	1.644	.186
ST * RD * PT	1.081	12	.090	4.403	.000
Error	1.636	80	.020		
Total	397.889	120			
Corrected Total	58.865	119			

a. R Squared = .972 (Adjusted R Squared = .959)

Estimated Marginal Means

1. Grand Mean

Dependent Variable:Fennel

Mean	Std. Error	95% Confidence Interval	
		Lower Bound	Upper Bound
1.681	.013	1.655	1.707

2. StorageTime

Estimates

Dependent Variable:Fennel

StorageTime	Mean	Std. Error	95% Confidence Interval	
			Lower Bound	Upper Bound
0 time	2.373	.029	2.315	2.431
12 mon	1.123	.029	1.065	1.181
3 mont	2.103	.029	2.045	2.161
6 mont	1.525	.029	1.467	1.583
9 mont	1.279	.029	1.221	1.337

Pairwise Comparisons

Dependent Variable:Fennel

(I) StorageTime	(J) StorageTime	Mean Difference (I-J)	Std. Error	Sig.[a]	95% Confidence Interval for Difference[a]	
					Lower Bound	Upper Bound
0 time	12 mon	1.250*	.041	.000	1.168	1.332
	3 mont	.270*	.041	.000	.188	.352
	6 mont	.848*	.041	.000	.766	.930
	9 mont	1.094*	.041	.000	1.012	1.176
12 mon	0 time	-1.250*	.041	.000	-1.332	-1.168
	3 mont	-.980*	.041	.000	-1.062	-.898
	6 mont	-.402*	.041	.000	-.484	-.320
	9 mont	-.156*	.041	.000	-.238	-.074
3 mont	0 time	-.270*	.041	.000	-.352	-.188
	12 mon	.980*	.041	.000	.898	1.062
	6 mont	.578*	.041	.000	.496	.660
	9 mont	.824*	.041	.000	.742	.906
6 mont	0 time	-.848*	.041	.000	-.930	-.766
	12 mon	.402*	.041	.000	.320	.484
	3 mont	-.578*	.041	.000	-.660	-.496
	9 mont	.246*	.041	.000	.164	.328
9 mont	0 time	-1.094*	.041	.000	-1.176	-1.012
	12 mon	.156*	.041	.000	.074	.238

	3 mont	-.824*	.041	.000	-.906	-.742
	6 mont	-.246*	.041	.000	-.328	-.164

Based on estimated marginal means

*. The mean difference is significant at the .05 level.

a. Adjustment for multiple comparisons: Least Significant Difference (equivalent to no adjustments).

Univariate Tests

Dependent Variable:Fennel

	Sum of Squares	df	Mean Square	F	Sig.
Contrast	27.708	4	6.927	338.670	.000
Error	1.636	80	.020		

The F tests the effect of StorageTime. This test is based on the linearly independent pairwise comparisons among the estimated marginal means.

3. Radiationdose

Estimates

Dependent Variable:Fennel

Radiationdose	Mean	Std. Error	95% Confidence Interval	
			Lower Bound	Upper Bound
0 UV	1.591	.018	1.554	1.627
UV	1.771	.018	1.734	1.808

Pairwise Comparisons

Dependent Variable:Fennel

(I) Radiationdose	(J) Radiationdose	Mean Difference (I-J)	Std. Error	Sig.[a]	95% Confidence Interval for Difference[a]	
					Lower Bound	Upper Bound
0 UV	UV	-.180*	.026	.000	-.232	-.128
UV	0 UV	.180*	.026	.000	.128	.232

Based on estimated marginal means

*. The mean difference is significant at the .05 level.

a. Adjustment for multiple comparisons: Least Significant Difference (equivalent to no adjustments).

Univariate Tests

Dependent Variable:Fennel

	Sum of Squares	df	Mean Square	F	Sig.
Contrast	.976	1	.976	47.699	.000
Error	1.636	80	.020		

The F tests the effect of Radiationdose. This test is based on the linearly independent pairwise comparisons among the estimated marginal means.

4. PackagingType

Estimates

Dependent Variable:Fennel

PackagingType	Mean	Std. Error	95% Confidence Interval	
			Lower Bound	Upper Bound
120	1.894	.026	1.842	1.946
150	1.915	.026	1.863	1.967
Anti	1.753	.026	1.701	1.805
Jute	1.161	.026	1.109	1.213

Pairwise Comparisons

Dependent Variable:Fennel

(I) PackagingType	(J) PackagingType	Mean Difference (I-J)	Std. Error	Sig.[a]	95% Confidence Interval for Difference[a]	
					Lower Bound	Upper Bound
120	150	-.021	.037	.577	-.094	.053
	Anti	.141*	.037	.000	.067	.214
	Jute	.733*	.037	.000	.659	.806
150	120	.021	.037	.577	-.053	.094
	Anti	.161*	.037	.000	.088	.235
	Jute	.753*	.037	.000	.680	.827
Anti	120	-.141*	.037	.000	-.214	-.067
	150	-.161*	.037	.000	-.235	-.088

	Jute	.592*	.037	.000	.519	.665
Jute	120	-.733*	.037	.000	-.806	-.659
	150	-.753*	.037	.000	-.827	-.680
	Anti	-.592*	.037	.000	-.665	-.519

Based on estimated marginal means

a. Adjustment for multiple comparisons: Least Significant Difference (equivalent to no adjustments).

*. The mean difference is significant at the .05 level.

Univariate Tests

Dependent Variable:Fennel

	Sum of Squares	df	Mean Square	F	Sig.
Contrast	11.258	3	3.753	183.469	.000
Error	1.636	80	.020		

The F tests the effect of PackagingType. This test is based on the linearly independent pairwise comparisons among the estimated marginal means.

5. StorageTime * Radiationdose

Dependent Variable:Fennel

StorageTime	Radiationdose	Mean	Std. Error	95% Confidence Interval	
				Lower Bound	Upper Bound
0 time	0 UV	2.000	.041	1.918	2.082
	UV	2.747	.041	2.665	2.829
12 mon	0 UV	1.113	.041	1.031	1.195
	UV	1.133	.041	1.051	1.215
3 mont	0 UV	2.073	.041	1.991	2.155
	UV	2.133	.041	2.051	2.215
6 mont	0 UV	1.542	.041	1.460	1.624
	UV	1.508	.041	1.426	1.590
9 mont	0 UV	1.225	.041	1.143	1.307
	UV	1.333	.041	1.251	1.415

6. StorageTime * PackagingType

Dependent Variable:Fennel

StorageTime	PackagingType	Mean	Std. Error	95% Confidence Interval	
				Lower Bound	Upper Bound
0 time	120	2.373	.058	2.257	2.490
	150	2.373	.058	2.257	2.490
	Anti	2.373	.058	2.257	2.490
	Jute	2.373	.058	2.257	2.490
12 mon	120	1.613	.058	1.497	1.730
	150	1.533	.058	1.417	1.650
	Anti	1.347	.058	1.230	1.463
	Jute	8.535E-16	.058	-.116	.116
3 mont	120	2.000	.058	1.884	2.116
	150	2.267	.058	2.150	2.383
	Anti	1.947	.058	1.830	2.063
	Jute	2.200	.058	2.084	2.316
6 mont	120	1.617	.058	1.500	1.733
	150	1.683	.058	1.567	1.800
	Anti	1.567	.058	1.450	1.683
	Jute	1.233	.058	1.117	1.350
9 mont	120	1.867	.058	1.750	1.983
	150	1.717	.058	1.600	1.833
	Anti	1.533	.058	1.417	1.650
	Jute	1.832E-15	.058	-.116	.116

7. Radiationdose * PackagingType

Dependent Variable:Fennel

Radiationdose	PackagingType	Mean	Std. Error	95% Confidence Interval	
				Lower Bound	Upper Bound
0 UV	120	1.843	.037	1.769	1.916
	150	1.785	.037	1.712	1.859
	Anti	1.676	.037	1.603	1.749
	Jute	1.059	.037	.985	1.132
UV	120	1.945	.037	1.872	2.019
	150	2.044	.037	1.971	2.117

Anti	1.831	.037	1.757	1.904
Jute	1.264	.037	1.191	1.337

8. StorageTime * Radiationdose * PackagingType

Dependent Variable:Fennel

StorageTime	Radiationdose	PackagingType	Mean	Std. Error	95% Confidence Interval	
					Lower Bound	Upper Bound
0 time	0 UV	120	2.000	.083	1.836	2.164
		150	2.000	.083	1.836	2.164
		Anti	2.000	.083	1.836	2.164
		Jute	2.000	.083	1.836	2.164
	UV	120	2.747	.083	2.582	2.911
		150	2.747	.083	2.582	2.911
		Anti	2.747	.083	2.582	2.911
		Jute	2.747	.083	2.582	2.911
12 mon	0 UV	120	1.653	.083	1.489	1.818
		150	1.493	.083	1.329	1.658
		Anti	1.307	.083	1.142	1.471
		Jute	-5.343E-16	.083	-.164	.164
	UV	120	1.573	.083	1.409	1.738
		150	1.573	.083	1.409	1.738
		Anti	1.387	.083	1.222	1.551
		Jute	2.241E-15	.083	-.164	.164
3 mont	0 UV	120	2.027	.083	1.862	2.191
		150	2.267	.083	2.102	2.431
		Anti	2.107	.083	1.942	2.271
		Jute	1.893	.083	1.729	2.058
	UV	120	1.973	.083	1.809	2.138
		150	2.267	.083	2.102	2.431
		Anti	1.787	.083	1.622	1.951
		Jute	2.507	.083	2.342	2.671
6 mont	0 UV	120	1.600	.083	1.436	1.764
		150	1.667	.083	1.502	1.831
		Anti	1.500	.083	1.336	1.664

		Jute	1.400	.083	1.236	1.564
	UV	120	1.633	.083	1.469	1.798
		150	1.700	.083	1.536	1.864
		Anti	1.633	.083	1.469	1.798
		Jute	1.067	.083	.902	1.231
9 mont	0 UV	120	1.933	.083	1.769	2.098
		150	1.500	.083	1.336	1.664
		Anti	1.467	.083	1.302	1.631
		Jute	-3.053E-15	.083	-.164	.164
	UV	120	1.800	.083	1.636	1.964
		150	1.933	.083	1.769	2.098
		Anti	1.600	.083	1.436	1.764
		Jute	6.717E-15	.083	-.164	.164

Post Hoc Tests

StorageTime

Multiple Comparisons

Dependent Variable:Fennel

	(I) StorageTime	(J) StorageTime	Mean Difference (I-J)	Std. Error	Sig.	95% Confidence Interval Lower Bound	95% Confidence Interval Upper Bound
Tukey HSD	0 time	12 mon	1.2500*	.04128	.000	1.1348	1.3652
		3 mont	.2700*	.04128	.000	.1548	.3852
		6 mont	.8483*	.04128	.000	.7331	.9636
		9 mont	1.0942*	.04128	.000	.9789	1.2094
	12 mon	0 time	-1.2500*	.04128	.000	-1.3652	-1.1348
		3 mont	-.9800*	.04128	.000	-1.0952	-.8648
		6 mont	-.4017*	.04128	.000	-.5169	-.2864
		9 mont	-.1558*	.04128	.003	-.2711	-.0406
	3 mont	0 time	-.2700*	.04128	.000	-.3852	-.1548
		12 mon	.9800*	.04128	.000	.8648	1.0952
		6 mont	.5783*	.04128	.000	.4631	.6936
		9 mont	.8242*	.04128	.000	.7089	.9394

	6 mont	0 time	-.8483*	.04128	.000	-.9636	-.7331
		12 mon	.4017*	.04128	.000	.2864	.5169
		3 mont	-.5783*	.04128	.000	-.6936	-.4631
		9 mont	.2458*	.04128	.000	.1306	.3611
	9 mont	0 time	-1.0942*	.04128	.000	-1.2094	-.9789
		12 mon	.1558*	.04128	.003	.0406	.2711
		3 mont	-.8242*	.04128	.000	-.9394	-.7089
		6 mont	-.2458*	.04128	.000	-.3611	-.1306

Based on observed means.

The error term is Mean Square(Error) = .020.

*. The mean difference is significant at the .05 level.

Homogeneous Subsets

Fennel

	StorageTime	N	Subset				
			1	2	3	4	5
Tukey HSD[a]	12 mon	24	1.1233				
	9 mont	24		1.2792			
	6 mont	24			1.5250		
	3 mont	24				2.1033	
	0 time	24					2.3733
	Sig.		1.000	1.000	1.000	1.000	1.000
Duncan[a]	12 mon	24	1.1233				
	9 mont	24		1.2792			
	6 mont	24			1.5250		
	3 mont	24				2.1033	
	0 time	24					2.3733
	Sig.		1.000	1.000	1.000	1.000	1.000

Means for groups in homogeneous subsets are displayed.

Based on observed means.

The error term is Mean Square(Error) = .020.

a. Uses Harmonic Mean Sample Size = 24.000.

PackagingType

Multiple Comparisons

Dependent Variable:Fennel

	(I) PackagingType	(J) PackagingType	Mean Difference (I-J)	Std. Error	Sig.	95% Confidence Interval Lower Bound	Upper Bound
Tukey HSD	120	150	-.0207	.03693	.944	-.1176	.0762
		Anti	.1407*	.03693	.002	.0438	.2376
		Jute	.7327*	.03693	.000	.6358	.8296
	150	120	.0207	.03693	.944	-.0762	.1176
		Anti	.1613*	.03693	.000	.0644	.2582
		Jute	.7533*	.03693	.000	.6564	.8502
	Anti	120	-.1407*	.03693	.002	-.2376	-.0438
		150	-.1613*	.03693	.000	-.2582	-.0644
		Jute	.5920*	.03693	.000	.4951	.6889
	Jute	120	-.7327*	.03693	.000	-.8296	-.6358
		150	-.7533*	.03693	.000	-.8502	-.6564
		Anti	-.5920*	.03693	.000	-.6889	-.4951

Based on observed means.

The error term is Mean Square(Error) = .020.

*. The mean difference is significant at the .05 level.

Homogeneous Subsets

Fennel

	PackagingType	N	Subset 1	2	3
Tukey HSD[a]	Jute	30	1.1613		
	Anti	30		1.7533	
	120	30			1.8940
	150	30			1.9147
	Sig.		1.000	1.000	.944
Duncan[a]	Jute	30	1.1613		

	Anti	30		1.7533	
	120	30			1.8940
	150	30			1.9147
	Sig.		1.000	1.000	.577

Means for groups in homogeneous subsets are displayed.

Based on observed means.

The error term is Mean Square(Error) = .020.

a. Uses Harmonic Mean Sample Size = 30.000.

```
UNIANOVA An BY ST RD PT
  /METHOD=SSTYPE(3)
  /INTERCEPT=INCLUDE
  /POSTHOC=ST RD PT(TUKEY DUNCAN)
  /EMMEANS=TABLES(OVERALL)
  /EMMEANS=TABLES(ST) COMPARE ADJ(LSD)
  /EMMEANS=TABLES(RD) COMPARE ADJ(LSD)
  /EMMEANS=TABLES(PT) COMPARE ADJ(LSD)
  /EMMEANS=TABLES(ST*RD)
  /EMMEANS=TABLES(ST*PT)
  /EMMEANS=TABLES(RD*PT)
  /EMMEANS=TABLES(ST*RD*PT)
  /PRINT=HOMOGENEITY DESCRIPTIVE
  /CRITERIA=ALPHA(.05)

  /DESIGN=ST RD PT ST*RD ST*PT RD*PT ST*RD*PT.
```

Univariate Analysis of Variance

Notes

Output Created		29-Nov-2025 08:32:29
Comments		
Input	Active Dataset	DataSet0
	Filter	<none>
	Weight	<none>
	Split File	<none>
	N of Rows in Working Data File	120
Missing Value Handling	Definition of Missing	User-defined missing values are treated as missing.
	Cases Used	Statistics are based on all cases with valid data for all variables in the model.

Syntax		UNIANOVA An BY ST RD PT<br>/METHOD=SSTYPE(3)<br>/INTERCEPT=INCLUDE<br>/POSTHOC=ST RD PT(TUKEY DUNCAN)<br>/EMMEANS=TABLES(OVERALL)<br>/EMMEANS=TABLES(ST) COMPARE ADJ(LSD)<br>/EMMEANS=TABLES(RD) COMPARE ADJ(LSD)<br>/EMMEANS=TABLES(PT) COMPARE ADJ(LSD)<br>/EMMEANS=TABLES(ST*RD)<br>/EMMEANS=TABLES(ST*PT)<br>/EMMEANS=TABLES(RD*PT)<br>/EMMEANS=TABLES(ST*RD*PT)<br>/PRINT=HOMOGENEITY DESCRIPTIVE<br>/CRITERIA=ALPHA(.05)<br>/DESIGN=ST RD PT ST*RD ST*PT RD*PT ST*RD*PT.
Resources	Processor Time	00:00:00.188
	Elapsed Time	00:00:00.078

[DataSet0]

Warnings

Post hoc tests are not performed for Radiationdose because there are fewer than three groups.

Between-Subjects Factors

		N
StorageTime	0 time	24
	12 mon	24
	3 mont	24
	6 mont	24
	9 mont	24

Radiationdose	0 UV	60
	UV	60
PackagingType	120	30
	150	30
	Anti	30
	Jute	30

Descriptive Statistics

Dependent Variable:Anise

StorageTime	Radiationdose	PackagingType	Mean	Std. Deviation	N
0 time	0 UV	120	2.8800	.21166	3
		150	2.8800	.21166	3
		Anti	2.8800	.21166	3
		Jute	2.8800	.21166	3
		Total	2.8800	.18050	12
	UV	120	3.0667	.24440	3
		150	3.0667	.24440	3
		Anti	3.0667	.24440	3
		Jute	3.0667	.24440	3
		Total	3.0667	.20843	12
	Total	120	2.9733	.22862	6
		150	2.9733	.22862	6
		Anti	2.9733	.22862	6
		Jute	2.9733	.22862	6
		Total	2.9733	.21319	24
12 mon	0 UV	120	1.8400	.63498	3
		150	2.0000	.21166	3
		Anti	1.8933	.12220	3
		Jute	.0000	.00000	3
		Total	1.4333	.91371	12
	UV	120	1.9200	.21166	3
		150	1.8133	.37807	3
		Anti	1.9200	.08000	3

		Jute	.0000	.00000	3
		Total	1.4133	.87392	12
	Total	120	1.8800	.42558	6
		150	1.9067	.29248	6
		Anti	1.9067	.09352	6
		Jute	.0000	.00000	6
		Total	1.4233	.87444	24
3 mont	0 UV	120	2.2667	.12220	3
		150	2.2933	.04619	3
		Anti	2.0133	.18903	3
		Jute	2.6133	.04619	3
		Total	2.2967	.24388	12
	UV	120	2.2400	.08000	3
		150	1.9467	.12220	3
		Anti	1.7867	.60044	3
		Jute	2.4800	.08000	3
		Total	2.1133	.38512	12
	Total	120	2.2533	.09352	6
		150	2.1200	.20707	6
		Anti	1.9000	.41704	6
		Jute	2.5467	.09352	6
		Total	2.2050	.32886	24
6 mont	0 UV	120	2.8333	.15275	3
		150	2.2000	.17321	3
		Anti	2.1333	.45092	3
		Jute	1.7000	.45826	3
		Total	2.2167	.51316	12
	UV	120	2.0667	.15275	3
		150	2.0000	.00000	3
		Anti	2.2000	.43589	3
		Jute	2.4000	.10000	3
		Total	2.1667	.25702	12
	Total	120	2.4500	.44159	6
		150	2.1000	.15492	6
		Anti	2.1667	.39833	6

		Jute	2.0500	.48477	6
		Total	2.1917	.39773	24
9 mont	0 UV	120	1.9667	.05774	3
		150	1.9000	.10000	3
		Anti	1.9333	.11547	3
		Jute	.0000	.00000	3
		Total	1.4500	.87750	12
	UV	120	2.0667	.15275	3
		150	1.8333	.05774	3
		Anti	2.2667	.20817	3
		Jute	.0000	.00000	3
		Total	1.5417	.95008	12
	Total	120	2.0167	.11690	6
		150	1.8667	.08165	6
		Anti	2.1000	.23664	6
		Jute	.0000	.00000	6
		Total	1.4958	.89563	24
Total	0 UV	120	2.3573	.51851	15
		150	2.2547	.38004	15
		Anti	2.1707	.43193	15
		Jute	1.4387	1.29585	15
		Total	2.0553	.82106	60
	UV	120	2.2720	.45019	15
		150	2.1320	.52032	15
		Anti	2.2480	.55419	15
		Jute	1.5893	1.36819	15
		Total	2.0603	.84110	60
	Total	120	2.3147	.47908	30
		150	2.1933	.45201	30
		Anti	2.2093	.48978	30
		Jute	1.5140	1.31157	30
		Total	2.0578	.82765	120

Levene's Test of Equality of Error Variances[a]

Dependent Variable:Anise

F	df1	df2	Sig.
3.399	39	80	.000

Tests the null hypothesis that the error variance of the dependent variable is equal across groups.

a. Design: Intercept + ST + RD + PT + ST * RD + ST * PT + RD * PT + ST * RD * PT

Tests of Between-Subjects Effects

Dependent Variable:Anise

Source	Type III Sum of Squares	df	Mean Square	F	Sig.
Corrected Model	76.905[a]	39	1.972	34.219	.000
Intercept	508.161	1	508.161	8.818E3	.000
ST	38.307	4	9.577	166.188	.000
RD	.001	1	.001	.013	.909
PT	12.091	3	4.030	69.938	.000
ST * RD	.478	4	.119	2.073	.092
ST * PT	24.078	12	2.006	34.819	.000
RD * PT	.382	3	.127	2.209	.093
ST * RD * PT	1.568	12	.131	2.268	.016
Error	4.610	80	.058		
Total	589.676	120			
Corrected Total	81.515	119			

a. R Squared = .943 (Adjusted R Squared = .916)

Estimated Marginal Means

1. Grand Mean

Dependent Variable:Anise

Mean	Std. Error	95% Confidence Interval	
		Lower Bound	Upper Bound
2.058	.022	2.014	2.101

2. StorageTime

Estimates

Dependent Variable:Anise

StorageTime	Mean	Std. Error	95% Confidence Interval	
			Lower Bound	Upper Bound
0 time	2.973	.049	2.876	3.071
12 mon	1.423	.049	1.326	1.521
3 mont	2.205	.049	2.107	2.303
6 mont	2.192	.049	2.094	2.289
9 mont	1.496	.049	1.398	1.593

Pairwise Comparisons

Dependent Variable:Anise

(I) StorageTime	(J) StorageTime	Mean Difference (I-J)	Std. Error	Sig.[a]	95% Confidence Interval for Difference[a]	
					Lower Bound	Upper Bound
0 time	12 mon	1.550*	.069	.000	1.412	1.688
	3 mont	.768*	.069	.000	.630	.906
	6 mont	.782*	.069	.000	.644	.920
	9 mont	1.478*	.069	.000	1.340	1.615
12 mon	0 time	-1.550*	.069	.000	-1.688	-1.412
	3 mont	-.782*	.069	.000	-.920	-.644
	6 mont	-.768*	.069	.000	-.906	-.630
	9 mont	-.072	.069	.299	-.210	.065
3 mont	0 time	-.768*	.069	.000	-.906	-.630
	12 mon	.782*	.069	.000	.644	.920
	6 mont	.013	.069	.848	-.125	.151
	9 mont	.709*	.069	.000	.571	.847
6 mont	0 time	-.782*	.069	.000	-.920	-.644
	12 mon	.768*	.069	.000	.630	.906
	3 mont	-.013	.069	.848	-.151	.125
	9 mont	.696*	.069	.000	.558	.834
9 mont	0 time	-1.478*	.069	.000	-1.615	-1.340
	12 mon	.072	.069	.299	-.065	.210

	3 mont	-.709*	.069	.000	-.847	-.571
	6 mont	-.696*	.069	.000	-.834	-.558

Based on estimated marginal means

*. The mean difference is significant at the .05 level.

a. Adjustment for multiple comparisons: Least Significant Difference (equivalent to no adjustments).

Univariate Tests

Dependent Variable:Anise

	Sum of Squares	df	Mean Square	F	Sig.
Contrast	38.307	4	9.577	166.188	.000
Error	4.610	80	.058		

The F tests the effect of StorageTime. This test is based on the linearly independent pairwise comparisons among the estimated marginal means.

3. Radiationdose

Estimates

Dependent Variable:Anise

Radiationdose	Mean	Std. Error	95% Confidence Interval	
			Lower Bound	Upper Bound
0 UV	2.055	.031	1.994	2.117
UV	2.060	.031	1.999	2.122

Pairwise Comparisons

Dependent Variable:Anise

(I) Radiationdose	(J) Radiationdose	Mean Difference (I-J)	Std. Error	Sig.[a]	95% Confidence Interval for Difference[a]	
					Lower Bound	Upper Bound
0 UV	UV	-.005	.044	.909	-.092	.082
UV	0 UV	.005	.044	.909	-.082	.092

Based on estimated marginal means

a. Adjustment for multiple comparisons: Least Significant Difference (equivalent to no adjustments).

Univariate Tests

Dependent Variable:Anise

	Sum of Squares	df	Mean Square	F	Sig.
Contrast	.001	1	.001	.013	.909
Error	4.610	80	.058		

The F tests the effect of Radiationdose. This test is based on the linearly independent pairwise comparisons among the estimated marginal means.

4. PackagingType

Estimates

Dependent Variable:Anise

PackagingType	Mean	Std. Error	95% Confidence Interval	
			Lower Bound	Upper Bound
120	2.315	.044	2.227	2.402
150	2.193	.044	2.106	2.281
Anti	2.209	.044	2.122	2.297
Jute	1.514	.044	1.427	1.601

Pairwise Comparisons

Dependent Variable:Anise

(I) PackagingType	(J) PackagingType	Mean Difference (I-J)	Std. Error	Sig.[a]	95% Confidence Interval for Difference[a]	
					Lower Bound	Upper Bound
120	150	.121	.062	.054	-.002	.245
	Anti	.105	.062	.093	-.018	.229
	Jute	.801*	.062	.000	.677	.924
150	120	-.121	.062	.054	-.245	.002
	Anti	-.016	.062	.797	-.139	.107
	Jute	.679*	.062	.000	.556	.803
Anti	120	-.105	.062	.093	-.229	.018
	150	.016	.062	.797	-.107	.139

	Jute	.695*	.062	.000	.572	.819
Jute	120	-.801*	.062	.000	-.924	-.677
	150	-.679*	.062	.000	-.803	-.556
	Anti	-.695*	.062	.000	-.819	-.572

Based on estimated marginal means

a. Adjustment for multiple comparisons: Least Significant Difference (equivalent to no adjustments).

*. The mean difference is significant at the .05 level.

Univariate Tests

Dependent Variable:Anise

	Sum of Squares	df	Mean Square	F	Sig.
Contrast	12.091	3	4.030	69.938	.000
Error	4.610	80	.058		

The F tests the effect of PackagingType. This test is based on the linearly independent pairwise comparisons among the estimated marginal means.

5. StorageTime * Radiationdose

Dependent Variable:Anise

StorageTime	Radiationdose	Mean	Std. Error	95% Confidence Interval	
				Lower Bound	Upper Bound
0 time	0 UV	2.880	.069	2.742	3.018
	UV	3.067	.069	2.929	3.205
12 mon	0 UV	1.433	.069	1.295	1.571
	UV	1.413	.069	1.275	1.551
3 mont	0 UV	2.297	.069	2.159	2.435
	UV	2.113	.069	1.975	2.251
6 mont	0 UV	2.217	.069	2.079	2.355
	UV	2.167	.069	2.029	2.305
9 mont	0 UV	1.450	.069	1.312	1.588
	UV	1.542	.069	1.404	1.680

6. StorageTime * PackagingType

Dependent Variable:Anise

StorageTime	PackagingType	Mean	Std. Error	95% Confidence Interval	
				Lower Bound	Upper Bound
0 time	120	2.973	.098	2.778	3.168
	150	2.973	.098	2.778	3.168
	Anti	2.973	.098	2.778	3.168
	Jute	2.973	.098	2.778	3.168
12 mon	120	1.880	.098	1.685	2.075
	150	1.907	.098	1.712	2.102
	Anti	1.907	.098	1.712	2.102
	Jute	1.923E-15	.098	-.195	.195
3 mont	120	2.253	.098	2.058	2.448
	150	2.120	.098	1.925	2.315
	Anti	1.900	.098	1.705	2.095
	Jute	2.547	.098	2.352	2.742
6 mont	120	2.450	.098	2.255	2.645
	150	2.100	.098	1.905	2.295
	Anti	2.167	.098	1.972	2.362
	Jute	2.050	.098	1.855	2.245
9 mont	120	2.017	.098	1.822	2.212
	150	1.867	.098	1.672	2.062
	Anti	2.100	.098	1.905	2.295
	Jute	-9.749E-16	.098	-.195	.195

7. Radiationdose * PackagingType

Dependent Variable:Anise

Radiationdose	PackagingType	Mean	Std. Error	95% Confidence Interval	
				Lower Bound	Upper Bound
0 UV	120	2.357	.062	2.234	2.481
	150	2.255	.062	2.131	2.378
	Anti	2.171	.062	2.047	2.294
	Jute	1.439	.062	1.315	1.562
UV	120	2.272	.062	2.149	2.395
	150	2.132	.062	2.009	2.255

	Anti	2.248	.062	2.125	2.371
	Jute	1.589	.062	1.466	1.713

8. StorageTime * Radiationdose * PackagingType

Dependent Variable:Anise

StorageTime	Radiationdose	PackagingType	Mean	Std. Error	95% Confidence Interval	
					Lower Bound	Upper Bound
0 time	0 UV	120	2.880	.139	2.604	3.156
		150	2.880	.139	2.604	3.156
		Anti	2.880	.139	2.604	3.156
		Jute	2.880	.139	2.604	3.156
	UV	120	3.067	.139	2.791	3.342
		150	3.067	.139	2.791	3.342
		Anti	3.067	.139	2.791	3.342
		Jute	3.067	.139	2.791	3.342
12 mon	0 UV	120	1.840	.139	1.564	2.116
		150	2.000	.139	1.724	2.276
		Anti	1.893	.139	1.618	2.169
		Jute	8.646E-16	.139	-.276	.276
	UV	120	1.920	.139	1.644	2.196
		150	1.813	.139	1.538	2.089
		Anti	1.920	.139	1.644	2.196
		Jute	2.981E-15	.139	-.276	.276
3 mont	0 UV	120	2.267	.139	1.991	2.542
		150	2.293	.139	2.018	2.569
		Anti	2.013	.139	1.738	2.289
		Jute	2.613	.139	2.338	2.889
	UV	120	2.240	.139	1.964	2.516
		150	1.947	.139	1.671	2.222
		Anti	1.787	.139	1.511	2.062
		Jute	2.480	.139	2.204	2.756
6 mont	0 UV	120	2.833	.139	2.558	3.109
		150	2.200	.139	1.924	2.476
		Anti	2.133	.139	1.858	2.409

		Jute	1.700	.139	1.424	1.976
	UV	120	2.067	.139	1.791	2.342
		150	2.000	.139	1.724	2.276
		Anti	2.200	.139	1.924	2.476
		Jute	2.400	.139	2.124	2.676
9 mont	0 UV	120	1.967	.139	1.691	2.242
		150	1.900	.139	1.624	2.176
		Anti	1.933	.139	1.658	2.209
		Jute	-2.748E-15	.139	-.276	.276
	UV	120	2.067	.139	1.791	2.342
		150	1.833	.139	1.558	2.109
		Anti	2.267	.139	1.991	2.542
		Jute	7.982E-16	.139	-.276	.276

Post Hoc Tests

StorageTime

Multiple Comparisons

Dependent Variable:Anise

	(I) StorageTime	(J) StorageTime	Mean Difference (I-J)	Std. Error	Sig.	95% Confidence Interval Lower Bound	Upper Bound
Tukey HSD	0 time	12 mon	1.5500*	.06930	.000	1.3566	1.7434
		3 mont	.7683*	.06930	.000	.5749	.9617
		6 mont	.7817*	.06930	.000	.5883	.9751
		9 mont	1.4775*	.06930	.000	1.2841	1.6709
	12 mon	0 time	-1.5500*	.06930	.000	-1.7434	-1.3566
		3 mont	-.7817*	.06930	.000	-.9751	-.5883
		6 mont	-.7683*	.06930	.000	-.9617	-.5749
		9 mont	-.0725	.06930	.833	-.2659	.1209
	3 mont	0 time	-.7683*	.06930	.000	-.9617	-.5749
		12 mon	.7817*	.06930	.000	.5883	.9751
		6 mont	.0133	.06930	1.000	-.1801	.2067
		9 mont	.7092*	.06930	.000	.5158	.9026

	6 mont	0 time	-.7817*	.06930	.000	-.9751	-.5883
		12 mon	.7683*	.06930	.000	.5749	.9617
		3 mont	-.0133	.06930	1.000	-.2067	.1801
		9 mont	.6958*	.06930	.000	.5024	.8892
	9 mont	0 time	-1.4775*	.06930	.000	-1.6709	-1.2841
		12 mon	.0725	.06930	.833	-.1209	.2659
		3 mont	-.7092*	.06930	.000	-.9026	-.5158
		6 mont	-.6958*	.06930	.000	-.8892	-.5024

Based on observed means.

The error term is Mean Square(Error) = .058.

*. The mean difference is significant at the .05 level.

Homogeneous Subsets

Anise

	StorageTime	N	Subset		
			1	2	3
Tukey HSD[a]	12 mon	24	1.4233		
	9 mont	24	1.4958		
	6 mont	24		2.1917	
	3 mont	24		2.2050	
	0 time	24			2.9733
	Sig.		.833	1.000	1.000
Duncan[a]	12 mon	24	1.4233		
	9 mont	24	1.4958		
	6 mont	24		2.1917	
	3 mont	24		2.2050	
	0 time	24			2.9733
	Sig.		.299	.848	1.000

Means for groups in homogeneous subsets are displayed.

Based on observed means.

The error term is Mean Square(Error) = .058.

a. Uses Harmonic Mean Sample Size = 24.000.

PackagingType

Multiple Comparisons

Dependent Variable:Anise

	(I) PackagingType	(J) PackagingType	Mean Difference (I-J)	Std. Error	Sig.	95% Confidence Interval Lower Bound	Upper Bound
Tukey HSD	120	150	.1213	.06198	.213	-.0413	.2840
		Anti	.1053	.06198	.331	-.0573	.2680
		Jute	.8007*	.06198	.000	.6380	.9633
	150	120	-.1213	.06198	.213	-.2840	.0413
		Anti	-.0160	.06198	.994	-.1786	.1466
		Jute	.6793*	.06198	.000	.5167	.8420
	Anti	120	-.1053	.06198	.331	-.2680	.0573
		150	.0160	.06198	.994	-.1466	.1786
		Jute	.6953*	.06198	.000	.5327	.8580
	Jute	120	-.8007*	.06198	.000	-.9633	-.6380
		150	-.6793*	.06198	.000	-.8420	-.5167
		Anti	-.6953*	.06198	.000	-.8580	-.5327

Based on observed means.

The error term is Mean Square(Error) = .058.

*. The mean difference is significant at the .05 level.

Homogeneous Subsets

Anise

	PackagingType	N	Subset 1	2
Tukey HSD[a]	Jute	30	1.5140	
	150	30		2.1933
	Anti	30		2.2093
	120	30		2.3147
	Sig.		1.000	.213
Duncan[a]	Jute	30	1.5140	

150	30		2.1933
Anti	30		2.2093
120	30		2.3147
Sig.		1.000	.067

Means for groups in homogeneous subsets are displayed.

Based on observed means.

The error term is Mean Square(Error) = .058.

a. Uses Harmonic Mean Sample Size = 30.000.

```
UNIANOVA Cu BY ST RD PT
  /METHOD=SSTYPE(3)
  /INTERCEPT=INCLUDE
  /POSTHOC=ST RD PT(TUKEY DUNCAN)
  /EMMEANS=TABLES(OVERALL)
  /EMMEANS=TABLES(ST) COMPARE ADJ(LSD)
  /EMMEANS=TABLES(RD) COMPARE ADJ(LSD)
  /EMMEANS=TABLES(PT) COMPARE ADJ(LSD)
  /EMMEANS=TABLES(ST*RD)
  /EMMEANS=TABLES(ST*PT)
  /EMMEANS=TABLES(RD*PT)
  /EMMEANS=TABLES(ST*RD*PT)
  /PRINT=HOMOGENEITY DESCRIPTIVE
  /CRITERIA=ALPHA(.05)

  /DESIGN=ST RD PT ST*RD ST*PT RD*PT ST*RD*PT.
```

Univariate Analysis of Variance

Notes

Output Created		29-Nov-2025 08:33:13
Comments		
Input	Active Dataset	DataSet0
	Filter	<none>
	Weight	<none>
	Split File	<none>
	N of Rows in Working Data File	120
Missing Value Handling	Definition of Missing	User-defined missing values are treated as missing.
	Cases Used	Statistics are based on all cases with valid data for all variables in the model.

Syntax		UNIANOVA Cu BY ST RD PT<br>/METHOD=SSTYPE(3)<br>/INTERCEPT=INCLUDE<br>/POSTHOC=ST RD PT(TUKEY DUNCAN)<br>/EMMEANS=TABLES(OVERALL)<br>/EMMEANS=TABLES(ST) COMPARE ADJ(LSD)<br>/EMMEANS=TABLES(RD) COMPARE ADJ(LSD)<br>/EMMEANS=TABLES(PT) COMPARE ADJ(LSD)<br>/EMMEANS=TABLES(ST*RD)<br>/EMMEANS=TABLES(ST*PT)<br>/EMMEANS=TABLES(RD*PT)<br>/EMMEANS=TABLES(ST*RD*PT)<br>/PRINT=HOMOGENEITY DESCRIPTIVE<br>/CRITERIA=ALPHA(.05)<br>/DESIGN=ST RD PT ST*RD ST*PT RD*PT ST*RD*PT.
Resources	Processor Time	00:00:00.281
	Elapsed Time	00:00:00.125

```
[DataSet0]
```

Warnings

Post hoc tests are not performed for Radiationdose because there are fewer than three groups.

Between-Subjects Factors

		N
StorageTime	0 time	24
	12 mon	24
	3 mont	24
	6 mont	24
	9 mont	24

Radiationdose	0 UV	60
	UV	60
PackagingType	120	30
	150	30
	Anti	30
	Jute	30

Descriptive Statistics

Dependent Variable:Cumin

StorageTime	Radiationdose	PackagingType	Mean	Std. Deviation	N
0 time	0 UV	120	3.4400	.36661	3
		150	3.4400	.36661	3
		Anti	3.4400	.36661	3
		Jute	3.4400	.36661	3
		Total	3.4400	.31264	12
	UV	120	4.0533	.81715	3
		150	4.0533	.81715	3
		Anti	4.0533	.81715	3
		Jute	4.0533	.81715	3
		Total	4.0533	.69687	12
	Total	120	3.7467	.65856	6
		150	3.7467	.65856	6
		Anti	3.7467	.65856	6
		Jute	3.7467	.65856	6
		Total	3.7467	.61411	24
12 mon	0 UV	120	2.1333	.12220	3
		150	2.2400	.16000	3
		Anti	2.3467	.12220	3
		Jute	.0000	.00000	3
		Total	1.6800	1.02109	12
	UV	120	2.2400	.08000	3
		150	2.3200	.13856	3
		Anti	2.8267	.09238	3

		Jute	.0000	.00000	3
		Total	1.8467	1.14082	12
	Total	120	2.1867	.10930	6
		150	2.2800	.14085	6
		Anti	2.5867	.28019	6
		Jute	.0000	.00000	6
		Total	1.7633	1.06223	24
3 mont	0 UV	120	2.9400	.02000	3
		150	2.8133	.02309	3
		Anti	3.0667	.23094	3
		Jute	3.8000	.20000	3
		Total	3.1550	.42092	12
	UV	120	2.3200	.13856	3
		150	2.4800	.08000	3
		Anti	2.1333	.04619	3
		Jute	2.5333	.16653	3
		Total	2.3667	.19133	12
	Total	120	2.6300	.35094	6
		150	2.6467	.19002	6
		Anti	2.6000	.53247	6
		Jute	3.1667	.71304	6
		Total	2.7608	.51416	24
6 mont	0 UV	120	2.5333	.07638	3
		150	2.6833	.07638	3
		Anti	2.7500	.21794	3
		Jute	2.9167	.48563	3
		Total	2.7208	.27258	12
	UV	120	3.0667	.11547	3
		150	3.2833	.24664	3
		Anti	3.0500	.13229	3
		Jute	3.2000	.17321	3
		Total	3.1500	.17965	12
	Total	120	2.8000	.30496	6
		150	2.9833	.36697	6
		Anti	2.9000	.23022	6

		Jute	3.0583	.36113	6
		Total	2.9354	.31467	24
9 mont	0 UV	120	2.6667	.11547	3
		150	2.4667	.11547	3
		Anti	2.5467	.09238	3
		Jute	.0000	.00000	3
		Total	1.9200	1.16294	12
	UV	120	2.8933	.10066	3
		150	2.6667	.04619	3
		Anti	2.6800	.06928	3
		Jute	.0000	.00000	3
		Total	2.0600	1.24702	12
	Total	120	2.7800	.15748	6
		150	2.5667	.13486	6
		Anti	2.6133	.10328	6
		Jute	.0000	.00000	6
		Total	1.9900	1.18138	24
Total	0 UV	120	2.7427	.47630	15
		150	2.7287	.44939	15
		Anti	2.8300	.44422	15
		Jute	2.0313	1.75799	15
		Total	2.5832	.99332	60
	UV	120	2.9147	.74739	15
		150	2.9607	.73633	15
		Anti	2.9487	.72450	15
		Jute	1.9573	1.75754	15
		Total	2.6953	1.14177	60
	Total	120	2.8287	.62196	30
		150	2.8447	.61087	30
		Anti	2.8893	.59355	30
		Jute	1.9943	1.72760	30
		Total	2.6393	1.06710	120

Levene's Test of Equality of Error Variances[a]

Dependent Variable:Cumin

F	df1	df2	Sig.
6.507	39	80	.000

Tests the null hypothesis that the error variance of the dependent variable is equal across groups.

a. Design: Intercept + ST + RD + PT + ST * RD + ST * PT + RD * PT + ST * RD * PT

Tests of Between-Subjects Effects

Dependent Variable:Cumin

Source	Type III Sum of Squares	df	Mean Square	F	Sig.
Corrected Model	127.673[a]	39	3.274	33.432	.000
Intercept	835.877	1	835.877	8.536E3	.000
ST	60.423	4	15.106	154.266	.000
RD	.377	1	.377	3.855	.053
PT	16.696	3	5.565	56.836	.000
ST * RD	6.998	4	1.749	17.866	.000
ST * PT	42.082	12	3.507	35.813	.000
RD * PT	.395	3	.132	1.344	.266
ST * RD * PT	.702	12	.058	.597	.838
Error	7.834	80	.098		
Total	971.384	120			
Corrected Total	135.507	119			

a. R Squared = .942 (Adjusted R Squared = .914)

Estimated Marginal Means

1. Grand Mean

Dependent Variable:Cumin

Mean	Std. Error	95% Confidence Interval	
		Lower Bound	Upper Bound
2.639	.029	2.582	2.696

2. StorageTime

Estimates

Dependent Variable:Cumin

StorageTime	Mean	Std. Error	95% Confidence Interval	
			Lower Bound	Upper Bound
0 time	3.747	.064	3.620	3.874
12 mon	1.763	.064	1.636	1.890
3 mont	2.761	.064	2.634	2.888
6 mont	2.935	.064	2.808	3.063
9 mont	1.990	.064	1.863	2.117

Pairwise Comparisons

Dependent Variable:Cumin

(I) StorageTime	(J) StorageTime	Mean Difference (I-J)	Std. Error	Sig.[a]	95% Confidence Interval for Difference[a]	
					Lower Bound	Upper Bound
0 time	12 mon	1.983*	.090	.000	1.804	2.163
	3 mont	.986*	.090	.000	.806	1.166
	6 mont	.811*	.090	.000	.631	.991
	9 mont	1.757*	.090	.000	1.577	1.936
12 mon	0 time	-1.983*	.090	.000	-2.163	-1.804
	3 mont	-.997*	.090	.000	-1.177	-.818
	6 mont	-1.172*	.090	.000	-1.352	-.992
	9 mont	-.227*	.090	.014	-.406	-.047
3 mont	0 time	-.986*	.090	.000	-1.166	-.806
	12 mon	.997*	.090	.000	.818	1.177
	6 mont	-.175	.090	.057	-.354	.005
	9 mont	.771*	.090	.000	.591	.951
6 mont	0 time	-.811*	.090	.000	-.991	-.631
	12 mon	1.172*	.090	.000	.992	1.352
	3 mont	.175	.090	.057	-.005	.354
	9 mont	.945*	.090	.000	.766	1.125
9 mont	0 time	-1.757*	.090	.000	-1.936	-1.577
	12 mon	.227*	.090	.014	.047	.406

	3 mont	-.771*	.090	.000	-.951	-.591
	6 mont	-.945*	.090	.000	-1.125	-.766

Based on estimated marginal means

*. The mean difference is significant at the .05 level.

a. Adjustment for multiple comparisons: Least Significant Difference (equivalent to no adjustments).

Univariate Tests

Dependent Variable:Cumin

	Sum of Squares	df	Mean Square	F	Sig.
Contrast	60.423	4	15.106	154.266	.000
Error	7.834	80	.098		

The F tests the effect of StorageTime. This test is based on the linearly independent pairwise comparisons among the estimated marginal means.

3. Radiationdose

Estimates

Dependent Variable:Cumin

Radiationdose	Mean	Std. Error	95% Confidence Interval	
			Lower Bound	Upper Bound
0 UV	2.583	.040	2.503	2.664
UV	2.695	.040	2.615	2.776

Pairwise Comparisons

Dependent Variable:Cumin

(I) Radiationdose	(J) Radiationdose	Mean Difference (I-J)	Std. Error	Sig.[a]	95% Confidence Interval for Difference[a]	
					Lower Bound	Upper Bound
0 UV	UV	-.112	.057	.053	-.226	.002
UV	0 UV	.112	.057	.053	-.002	.226

Based on estimated marginal means

a. Adjustment for multiple comparisons: Least Significant Difference (equivalent to no adjustments).

Univariate Tests

Dependent Variable:Cumin

	Sum of Squares	df	Mean Square	F	Sig.
Contrast	.377	1	.377	3.855	.053
Error	7.834	80	.098		

The F tests the effect of Radiationdose. This test is based on the linearly independent pairwise comparisons among the estimated marginal means.

4. PackagingType

Estimates

Dependent Variable:Cumin

PackagingType	Mean	Std. Error	95% Confidence Interval	
			Lower Bound	Upper Bound
120	2.829	.057	2.715	2.942
150	2.845	.057	2.731	2.958
Anti	2.889	.057	2.776	3.003
Jute	1.994	.057	1.881	2.108

Pairwise Comparisons

Dependent Variable:Cumin

(I) PackagingType	(J) PackagingType	Mean Difference (I-J)	Std. Error	Sig.[a]	95% Confidence Interval for Difference[a]	
					Lower Bound	Upper Bound
120	150	-.016	.081	.844	-.177	.145
	Anti	-.061	.081	.455	-.221	.100
	Jute	.834*	.081	.000	.674	.995
150	120	.016	.081	.844	-.145	.177
	Anti	-.045	.081	.582	-.205	.116
	Jute	.850*	.081	.000	.690	1.011
Anti	120	.061	.081	.455	-.100	.221
	150	.045	.081	.582	-.116	.205

	Jute	.895*	.081	.000	.734	1.056
Jute	120	-.834*	.081	.000	-.995	-.674
	150	-.850*	.081	.000	-1.011	-.690
	Anti	-.895*	.081	.000	-1.056	-.734

Based on estimated marginal means

a. Adjustment for multiple comparisons: Least Significant Difference (equivalent to no adjustments).

*. The mean difference is significant at the .05 level.

Univariate Tests

Dependent Variable:Cumin

	Sum of Squares	df	Mean Square	F	Sig.
Contrast	16.696	3	5.565	56.836	.000
Error	7.834	80	.098		

The F tests the effect of PackagingType. This test is based on the linearly independent pairwise comparisons among the estimated marginal means.

5. StorageTime * Radiationdose

Dependent Variable:Cumin

StorageTime	Radiationdose	Mean	Std. Error	95% Confidence Interval	
				Lower Bound	Upper Bound
0 time	0 UV	3.440	.090	3.260	3.620
	UV	4.053	.090	3.874	4.233
12 mon	0 UV	1.680	.090	1.500	1.860
	UV	1.847	.090	1.667	2.026
3 mont	0 UV	3.155	.090	2.975	3.335
	UV	2.367	.090	2.187	2.546
6 mont	0 UV	2.721	.090	2.541	2.901
	UV	3.150	.090	2.970	3.330
9 mont	0 UV	1.920	.090	1.740	2.100
	UV	2.060	.090	1.880	2.240

6. StorageTime * PackagingType

Dependent Variable:Cumin

StorageTime	PackagingType	Mean	Std. Error	95% Confidence Interval	
				Lower Bound	Upper Bound
0 time	120	3.747	.128	3.492	4.001
	150	3.747	.128	3.492	4.001
	Anti	3.747	.128	3.492	4.001
	Jute	3.747	.128	3.492	4.001
12 mon	120	2.187	.128	1.932	2.441
	150	2.280	.128	2.026	2.534
	Anti	2.587	.128	2.332	2.841
	Jute	1.850E-15	.128	-.254	.254
3 mont	120	2.630	.128	2.376	2.884
	150	2.647	.128	2.392	2.901
	Anti	2.600	.128	2.346	2.854
	Jute	3.167	.128	2.912	3.421
6 mont	120	2.800	.128	2.546	3.054
	150	2.983	.128	2.729	3.238
	Anti	2.900	.128	2.646	3.154
	Jute	3.058	.128	2.804	3.313
9 mont	120	2.780	.128	2.526	3.034
	150	2.567	.128	2.312	2.821
	Anti	2.613	.128	2.359	2.868
	Jute	2.591E-14	.128	-.254	.254

7. Radiationdose * PackagingType

Dependent Variable:Cumin

Radiationdose	PackagingType	Mean	Std. Error	95% Confidence Interval	
				Lower Bound	Upper Bound
0 UV	120	2.743	.081	2.582	2.903
	150	2.729	.081	2.568	2.889
	Anti	2.830	.081	2.669	2.991
	Jute	2.031	.081	1.871	2.192
UV	120	2.915	.081	2.754	3.075
	150	2.961	.081	2.800	3.121

Anti	2.949	.081	2.788	3.109
Jute	1.957	.081	1.797	2.118

8. StorageTime * Radiationdose * PackagingType

Dependent Variable:Cumin

StorageTime	Radiationdose	PackagingType	Mean	Std. Error	95% Confidence Interval Lower Bound	Upper Bound
0 time	0 UV	120	3.440	.181	3.080	3.800
		150	3.440	.181	3.080	3.800
		Anti	3.440	.181	3.080	3.800
		Jute	3.440	.181	3.080	3.800
	UV	120	4.053	.181	3.694	4.413
		150	4.053	.181	3.694	4.413
		Anti	4.053	.181	3.694	4.413
		Jute	4.053	.181	3.694	4.413
12 mon	0 UV	120	2.133	.181	1.774	2.493
		150	2.240	.181	1.880	2.600
		Anti	2.347	.181	1.987	2.706
		Jute	3.410E-16	.181	-.360	.360
	UV	120	2.240	.181	1.880	2.600
		150	2.320	.181	1.960	2.680
		Anti	2.827	.181	2.467	3.186
		Jute	3.359E-15	.181	-.360	.360
3 mont	0 UV	120	2.940	.181	2.580	3.300
		150	2.813	.181	2.454	3.173
		Anti	3.067	.181	2.707	3.426
		Jute	3.800	.181	3.440	4.160
	UV	120	2.320	.181	1.960	2.680
		150	2.480	.181	2.120	2.840
		Anti	2.133	.181	1.774	2.493
		Jute	2.533	.181	2.174	2.893
6 mont	0 UV	120	2.533	.181	2.174	2.893
		150	2.683	.181	2.324	3.043
		Anti	2.750	.181	2.390	3.110

		Jute	2.917	.181	2.557	3.276
	UV	120	3.067	.181	2.707	3.426
		150	3.283	.181	2.924	3.643
		Anti	3.050	.181	2.690	3.410
		Jute	3.200	.181	2.840	3.560
9 mont	0 UV	120	2.667	.181	2.307	3.026
		150	2.467	.181	2.107	2.826
		Anti	2.547	.181	2.187	2.906
		Jute	2.894E-15	.181	-.360	.360
	UV	120	2.893	.181	2.534	3.253
		150	2.667	.181	2.307	3.026
		Anti	2.680	.181	2.320	3.040
		Jute	4.893E-14	.181	-.360	.360

Post Hoc Tests

StorageTime

Multiple Comparisons

Dependent Variable:Cumin

	(I) StorageTime	(J) StorageTime	Mean Difference (I-J)	Std. Error	Sig.	95% Confidence Interval Lower Bound	Upper Bound
Tukey HSD	0 time	12 mon	1.9833*	.09033	.000	1.7312	2.2354
		3 mont	.9858*	.09033	.000	.7337	1.2379
		6 mont	.8112*	.09033	.000	.5591	1.0634
		9 mont	1.7567*	.09033	.000	1.5046	2.0088
	12 mon	0 time	-1.9833*	.09033	.000	-2.2354	-1.7312
		3 mont	-.9975*	.09033	.000	-1.2496	-.7454
		6 mont	-1.1721*	.09033	.000	-1.4242	-.9200
		9 mont	-.2267	.09033	.099	-.4788	.0254
	3 mont	0 time	-.9858*	.09033	.000	-1.2379	-.7337
		12 mon	.9975*	.09033	.000	.7454	1.2496
		6 mont	-.1746	.09033	.309	-.4267	.0775
		9 mont	.7708*	.09033	.000	.5187	1.0229

	6 mont	0 time	-.8112*	.09033	.000	-1.0634	-.5591
		12 mon	1.1721*	.09033	.000	.9200	1.4242
		3 mont	.1746	.09033	.309	-.0775	.4267
		9 mont	.9454*	.09033	.000	.6933	1.1975
	9 mont	0 time	-1.7567*	.09033	.000	-2.0088	-1.5046
		12 mon	.2267	.09033	.099	-.0254	.4788
		3 mont	-.7708*	.09033	.000	-1.0229	-.5187
		6 mont	-.9454*	.09033	.000	-1.1975	-.6933

Based on observed means.

The error term is Mean Square(Error) = .098.

*. The mean difference is significant at the .05 level.

Homogeneous Subsets

Cumin

	StorageTime	N	Subset			
			1	2	3	4
Tukey HSD[a]	12 mon	24	1.7633			
	9 mont	24	1.9900			
	3 mont	24		2.7608		
	6 mont	24		2.9354		
	0 time	24			3.7467	
	Sig.		.099	.309	1.000	
Duncan[a]	12 mon	24	1.7633			
	9 mont	24		1.9900		
	3 mont	24			2.7608	
	6 mont	24			2.9354	
	0 time	24				3.7467
	Sig.		1.000	1.000	.057	1.000

Means for groups in homogeneous subsets are displayed.

Based on observed means.

The error term is Mean Square(Error) = .098.

a. Uses Harmonic Mean Sample Size = 24.000.

PackagingType

Multiple Comparisons

Dependent Variable:Cumin

	(I) PackagingType	(J) PackagingType	Mean Difference (I-J)	Std. Error	Sig.	95% Confidence Interval Lower Bound	Upper Bound
Tukey HSD	120	150	-.0160	.08080	.997	-.2280	.1960
		Anti	-.0607	.08080	.876	-.2727	.1513
		Jute	.8343*	.08080	.000	.6223	1.0463
	150	120	.0160	.08080	.997	-.1960	.2280
		Anti	-.0447	.08080	.946	-.2567	.1673
		Jute	.8503*	.08080	.000	.6383	1.0623
	Anti	120	.0607	.08080	.876	-.1513	.2727
		150	.0447	.08080	.946	-.1673	.2567
		Jute	.8950*	.08080	.000	.6830	1.1070
	Jute	120	-.8343*	.08080	.000	-1.0463	-.6223
		150	-.8503*	.08080	.000	-1.0623	-.6383
		Anti	-.8950*	.08080	.000	-1.1070	-.6830

Based on observed means.

The error term is Mean Square(Error) = .098.

*. The mean difference is significant at the .05 level.

Homogeneous Subsets

Cumin

	PackagingType	N	Subset 1	2
Tukey HSD[a]	Jute	30	1.9943	
	120	30		2.8287
	150	30		2.8447
	Anti	30		2.8893
	Sig.		1.000	.876
Duncan[a]	Jute	30	1.9943	

120	30		2.8287
150	30		2.8447
Anti	30		2.8893
Sig.		1.000	.484

Means for groups in homogeneous subsets are displayed.

Based on observed means.

The error term is Mean Square(Error) = .098.

a. Uses Harmonic Mean Sample Size = 30.000.

```
UNIANOVA Ca BY ST RD PT
  /METHOD=SSTYPE(3)
  /INTERCEPT=INCLUDE
  /POSTHOC=ST RD PT(TUKEY DUNCAN)
  /EMMEANS=TABLES(OVERALL)
  /EMMEANS=TABLES(ST) COMPARE ADJ(LSD)
  /EMMEANS=TABLES(RD) COMPARE ADJ(LSD)
  /EMMEANS=TABLES(PT) COMPARE ADJ(LSD)
  /EMMEANS=TABLES(ST*RD)
  /EMMEANS=TABLES(ST*PT)
  /EMMEANS=TABLES(RD*PT)
  /EMMEANS=TABLES(ST*RD*PT)
  /PRINT=HOMOGENEITY DESCRIPTIVE
  /CRITERIA=ALPHA(.05)

  /DESIGN=ST RD PT ST*RD ST*PT RD*PT ST*RD*PT.
```

Univariate Analysis of Variance

Notes

Output Created		29-Nov-2025 08:33:28
Comments		
Input	Active Dataset	DataSet0
	Filter	<none>
	Weight	<none>
	Split File	<none>
	N of Rows in Working Data File	120
Missing Value Handling	Definition of Missing	User-defined missing values are treated as missing.
	Cases Used	Statistics are based on all cases with valid data for all variables in the model.

Syntax		UNIANOVA Ca BY ST RD PT<br>/METHOD=SSTYPE(3)<br>/INTERCEPT=INCLUDE<br>/POSTHOC=ST RD PT(TUKEY DUNCAN)<br>/EMMEANS=TABLES(OVERALL)<br>/EMMEANS=TABLES(ST) COMPARE ADJ(LSD)<br>/EMMEANS=TABLES(RD) COMPARE ADJ(LSD)<br>/EMMEANS=TABLES(PT) COMPARE ADJ(LSD)<br>/EMMEANS=TABLES(ST*RD)<br>/EMMEANS=TABLES(ST*PT)<br>/EMMEANS=TABLES(RD*PT)<br>/EMMEANS=TABLES(ST*RD*PT)<br>/PRINT=HOMOGENEITY DESCRIPTIVE<br>/CRITERIA=ALPHA(.05)<br>/DESIGN=ST RD PT ST*RD ST*PT RD*PT ST*RD*PT.
Resources	Processor Time	00:00:00.328
	Elapsed Time	00:00:00.142

[DataSet0]

Warnings

Post hoc tests are not performed for Radiationdose because there are fewer than three groups.

Between-Subjects Factors

		N
StorageTime	0 time	24
	12 mon	24
	3 mont	24
	6 mont	24
	9 mont	24

Radiationdose	0 UV	60
	UV	60
PackagingType	120	30
	150	30
	Anti	30
	Jute	30

Descriptive Statistics

Dependent Variable:Caraway

StorageTime	Radiationdose	PackagingType	Mean	Std. Deviation	N
0 time	0 UV	120	2.0533	.24440	3
		150	2.0533	.24440	3
		Anti	2.0533	.24440	3
		Jute	2.0533	.24440	3
		Total	2.0533	.20843	12
	UV	120	2.4533	.48222	3
		150	2.4533	.48222	3
		Anti	2.4533	.48222	3
		Jute	2.4533	.48222	3
		Total	2.4533	.41124	12
	Total	120	2.2533	.40609	6
		150	2.2533	.40609	6
		Anti	2.2533	.40609	6
		Jute	2.2533	.40609	6
		Total	2.2533	.37868	24
12 mon	0 UV	120	1.6000	.00000	3
		150	1.6000	.00000	3
		Anti	1.8667	.23094	3
		Jute	.0000	.00000	3
		Total	1.2667	.77850	12
	UV	120	1.9467	.18475	3
		150	2.0267	.09238	3
		Anti	2.1067	.09238	3

		Jute	.0000	.00000	3
		Total	1.5200	.92355	12
	Total	120	1.7733	.22295	6
		150	1.8133	.24089	6
		Anti	1.9867	.20500	6
		Jute	.0000	.00000	6
		Total	1.3933	.84530	24
3 mont	0 UV	120	1.2800	.08000	3
		150	1.3867	.20133	3
		Anti	1.2533	.46876	3
		Jute	1.2800	.08000	3
		Total	1.3000	.22915	12
	UV	120	1.7600	.13856	3
		150	2.2933	.25716	3
		Anti	1.5733	.28095	3
		Jute	1.8400	.34871	3
		Total	1.8667	.35831	12
	Total	120	1.5200	.28171	6
		150	1.8400	.53785	6
		Anti	1.4133	.38754	6
		Jute	1.5600	.38116	6
		Total	1.5833	.41265	24
6 mont	0 UV	120	1.6333	.15275	3
		150	1.7000	.10000	3
		Anti	1.4000	.20000	3
		Jute	1.5333	.11547	3
		Total	1.5667	.17233	12
	UV	120	1.5000	.43589	3
		150	1.8667	.11547	3
		Anti	1.7333	.25166	3
		Jute	1.5000	.10000	3
		Total	1.6500	.27798	12
	Total	120	1.5667	.30111	6
		150	1.7833	.13292	6
		Anti	1.5667	.27325	6

		Jute	1.5167	.09832	6
		Total	1.6083	.23015	24
9 mont	0 UV	120	2.0333	.15275	3
		150	1.6667	.11547	3
		Anti	1.7667	.05774	3
		Jute	.0000	.00000	3
		Total	1.3667	.84027	12
	UV	120	1.9333	.11547	3
		150	2.4333	.23094	3
		Anti	2.1333	.15275	3
		Jute	.0000	.00000	3
		Total	1.6250	1.00555	12
	Total	120	1.9833	.13292	6
		150	2.0500	.45056	6
		Anti	1.9500	.22583	6
		Jute	.0000	.00000	6
		Total	1.4958	.91579	24
Total	0 UV	120	1.7200	.32733	15
		150	1.6813	.25972	15
		Anti	1.6680	.38514	15
		Jute	.9733	.86872	15
		Total	1.5107	.59493	60
	UV	120	1.9187	.41751	15
		150	2.2147	.33273	15
		Anti	2.0000	.40327	15
		Jute	1.1587	1.05404	15
		Total	1.8230	.72894	60
	Total	120	1.8193	.38221	30
		150	1.9480	.39947	30
		Anti	1.8340	.42264	30
		Jute	1.0660	.95370	30
		Total	1.6668	.68082	120

Levene's Test of Equality of Error Variances[a]

Dependent Variable:Caraway

F	df1	df2	Sig.
2.850	39	80	.000

Tests the null hypothesis that the error variance of the dependent variable is equal across groups.

a. Design: Intercept + ST + RD + PT + ST * RD + ST * PT + RD * PT + ST * RD * PT

Tests of Between-Subjects Effects

Dependent Variable:Caraway

Source	Type III Sum of Squares	df	Mean Square	F	Sig.
Corrected Model	50.507[a]	39	1.295	22.272	.000
Intercept	333.400	1	333.400	5.734E3	.000
ST	11.002	4	2.751	47.303	.000
RD	2.927	1	2.927	50.331	.000
PT	14.738	3	4.913	84.486	.000
ST * RD	.787	4	.197	3.385	.013
ST * PT	19.730	12	1.644	28.276	.000
RD * PT	.587	3	.196	3.365	.023
ST * RD * PT	.737	12	.061	1.056	.408
Error	4.652	80	.058		
Total	388.559	120			
Corrected Total	55.159	119			

a. R Squared = .916 (Adjusted R Squared = .875)

Estimated Marginal Means

1. Grand Mean

Dependent Variable:Caraway

Mean	Std. Error	95% Confidence Interval	
		Lower Bound	Upper Bound
1.667	.022	1.623	1.711

2. StorageTime

Estimates

Dependent Variable:Caraway

StorageTime	Mean	Std. Error	95% Confidence Interval	
			Lower Bound	Upper Bound
0 time	2.253	.049	2.155	2.351
12 mon	1.393	.049	1.295	1.491
3 mont	1.583	.049	1.485	1.681
6 mont	1.608	.049	1.510	1.706
9 mont	1.496	.049	1.398	1.594

Pairwise Comparisons

Dependent Variable:Caraway

(I) StorageTime	(J) StorageTime	Mean Difference (I-J)	Std. Error	Sig.[a]	95% Confidence Interval for Difference[a]	
					Lower Bound	Upper Bound
0 time	12 mon	.860*	.070	.000	.721	.999
	3 mont	.670*	.070	.000	.531	.809
	6 mont	.645*	.070	.000	.506	.784
	9 mont	.757*	.070	.000	.619	.896
12 mon	0 time	-.860*	.070	.000	-.999	-.721
	3 mont	-.190*	.070	.008	-.329	-.051
	6 mont	-.215*	.070	.003	-.354	-.076
	9 mont	-.102	.070	.145	-.241	.036
3 mont	0 time	-.670*	.070	.000	-.809	-.531
	12 mon	.190*	.070	.008	.051	.329
	6 mont	-.025	.070	.720	-.164	.114
	9 mont	.088	.070	.212	-.051	.226
6 mont	0 time	-.645*	.070	.000	-.784	-.506
	12 mon	.215*	.070	.003	.076	.354
	3 mont	.025	.070	.720	-.114	.164
	9 mont	.113	.070	.110	-.026	.251
9 mont	0 time	-.757*	.070	.000	-.896	-.619
	12 mon	.102	.070	.145	-.036	.241

	3 mont	-.088	.070	.212	-.226	.051
	6 mont	-.113	.070	.110	-.251	.026

Based on estimated marginal means

*. The mean difference is significant at the .05 level.

a. Adjustment for multiple comparisons: Least Significant Difference (equivalent to no adjustments).

Univariate Tests

Dependent Variable:Caraway

	Sum of Squares	df	Mean Square	F	Sig.
Contrast	11.002	4	2.751	47.303	.000
Error	4.652	80	.058		

The F tests the effect of StorageTime. This test is based on the linearly independent pairwise comparisons among the estimated marginal means.

3. Radiationdose

Estimates

Dependent Variable:Caraway

Radiationdose	Mean	Std. Error	95% Confidence Interval	
			Lower Bound	Upper Bound
0 UV	1.511	.031	1.449	1.573
UV	1.823	.031	1.761	1.885

Pairwise Comparisons

Dependent Variable:Caraway

(I) Radiationdose	(J) Radiationdose	Mean Difference (I-J)	Std. Error	Sig.[a]	95% Confidence Interval for Difference[a]	
					Lower Bound	Upper Bound
0 UV	UV	-.312*	.044	.000	-.400	-.225
UV	0 UV	.312*	.044	.000	.225	.400

Based on estimated marginal means

*. The mean difference is significant at the .05 level.

a. Adjustment for multiple comparisons: Least Significant Difference (equivalent to no adjustments).

Univariate Tests

Dependent Variable:Caraway

	Sum of Squares	df	Mean Square	F	Sig.
Contrast	2.927	1	2.927	50.331	.000
Error	4.652	80	.058		

The F tests the effect of Radiationdose. This test is based on the linearly independent pairwise comparisons among the estimated marginal means.

4. PackagingType

Estimates

Dependent Variable:Caraway

PackagingType	Mean	Std. Error	95% Confidence Interval	
			Lower Bound	Upper Bound
120	1.819	.044	1.732	1.907
150	1.948	.044	1.860	2.036
Anti	1.834	.044	1.746	1.922
Jute	1.066	.044	.978	1.154

Pairwise Comparisons

Dependent Variable:Caraway

(I) PackagingType	(J) PackagingType	Mean Difference (I-J)	Std. Error	Sig.[a]	95% Confidence Interval for Difference[a]	
					Lower Bound	Upper Bound
120	150	-.129*	.062	.042	-.253	-.005
	Anti	-.015	.062	.814	-.139	.109
	Jute	.753*	.062	.000	.629	.877
150	120	.129*	.062	.042	.005	.253
	Anti	.114	.062	.071	-.010	.238
	Jute	.882*	.062	.000	.758	1.006
Anti	120	.015	.062	.814	-.109	.139
	150	-.114	.062	.071	-.238	.010

	Jute	.768*	.062	.000	.644	.892
Jute	120	-.753*	.062	.000	-.877	-.629
	150	-.882*	.062	.000	-1.006	-.758
	Anti	-.768*	.062	.000	-.892	-.644

Based on estimated marginal means

*. The mean difference is significant at the .05 level.

a. Adjustment for multiple comparisons: Least Significant Difference (equivalent to no adjustments).

Univariate Tests

Dependent Variable:Caraway

	Sum of Squares	df	Mean Square	F	Sig.
Contrast	14.738	3	4.913	84.486	.000
Error	4.652	80	.058		

The F tests the effect of PackagingType. This test is based on the linearly independent pairwise comparisons among the estimated marginal means.

5. StorageTime * Radiationdose

Dependent Variable:Caraway

StorageTime	Radiationdose	Mean	Std. Error	95% Confidence Interval	
				Lower Bound	Upper Bound
0 time	0 UV	2.053	.070	1.915	2.192
	UV	2.453	.070	2.315	2.592
12 mon	0 UV	1.267	.070	1.128	1.405
	UV	1.520	.070	1.381	1.659
3 mont	0 UV	1.300	.070	1.161	1.439
	UV	1.867	.070	1.728	2.005
6 mont	0 UV	1.567	.070	1.428	1.705
	UV	1.650	.070	1.511	1.789
9 mont	0 UV	1.367	.070	1.228	1.505
	UV	1.625	.070	1.486	1.764

6. StorageTime * PackagingType

Dependent Variable:Caraway

StorageTime	PackagingType	Mean	Std. Error	95% Confidence Interval	
				Lower Bound	Upper Bound
0 time	120	2.253	.098	2.057	2.449
	150	2.253	.098	2.057	2.449
	Anti	2.253	.098	2.057	2.449
	Jute	2.253	.098	2.057	2.449
12 mon	120	1.773	.098	1.577	1.969
	150	1.813	.098	1.617	2.009
	Anti	1.987	.098	1.791	2.183
	Jute	-2.554E-16	.098	-.196	.196
3 mont	120	1.520	.098	1.324	1.716
	150	1.840	.098	1.644	2.036
	Anti	1.413	.098	1.217	1.609
	Jute	1.560	.098	1.364	1.756
6 mont	120	1.567	.098	1.371	1.763
	150	1.783	.098	1.587	1.979
	Anti	1.567	.098	1.371	1.763
	Jute	1.517	.098	1.321	1.713
9 mont	120	1.983	.098	1.787	2.179
	150	2.050	.098	1.854	2.246
	Anti	1.950	.098	1.754	2.146
	Jute	-3.835E-15	.098	-.196	.196

7. Radiationdose * PackagingType

Dependent Variable:Caraway

Radiationdose	PackagingType	Mean	Std. Error	95% Confidence Interval	
				Lower Bound	Upper Bound
0 UV	120	1.720	.062	1.596	1.844
	150	1.681	.062	1.557	1.805
	Anti	1.668	.062	1.544	1.792
	Jute	.973	.062	.849	1.097
UV	120	1.919	.062	1.795	2.043
	150	2.215	.062	2.091	2.339

Anti	2.000	.062	1.876	2.124
Jute	1.159	.062	1.035	1.283

8. StorageTime * Radiationdose * PackagingType

Dependent Variable:Caraway

StorageTime	Radiationdose	PackagingType	Mean	Std. Error	95% Confidence Interval	
					Lower Bound	Upper Bound
0 time	0 UV	120	2.053	.139	1.776	2.330
		150	2.053	.139	1.776	2.330
		Anti	2.053	.139	1.776	2.330
		Jute	2.053	.139	1.776	2.330
	UV	120	2.453	.139	2.176	2.730
		150	2.453	.139	2.176	2.730
		Anti	2.453	.139	2.176	2.730
		Jute	2.453	.139	2.176	2.730
12 mon	0 UV	120	1.600	.139	1.323	1.877
		150	1.600	.139	1.323	1.877
		Anti	1.867	.139	1.590	2.144
		Jute	-3.099E-16	.139	-.277	.277
	UV	120	1.947	.139	1.670	2.224
		150	2.027	.139	1.750	2.304
		Anti	2.107	.139	1.830	2.384
		Jute	-2.008E-16	.139	-.277	.277
3 mont	0 UV	120	1.280	.139	1.003	1.557
		150	1.387	.139	1.110	1.664
		Anti	1.253	.139	.976	1.530
		Jute	1.280	.139	1.003	1.557
	UV	120	1.760	.139	1.483	2.037
		150	2.293	.139	2.016	2.570
		Anti	1.573	.139	1.296	1.850
		Jute	1.840	.139	1.563	2.117
6 mont	0 UV	120	1.633	.139	1.356	1.910
		150	1.700	.139	1.423	1.977
		Anti	1.400	.139	1.123	1.677

		Jute	1.533	.139	1.256	1.810
	UV	120	1.500	.139	1.223	1.777
		150	1.867	.139	1.590	2.144
		Anti	1.733	.139	1.456	2.010
		Jute	1.500	.139	1.223	1.777
9 mont	0 UV	120	2.033	.139	1.756	2.310
		150	1.667	.139	1.390	1.944
		Anti	1.767	.139	1.490	2.044
		Jute	-1.580E-14	.139	-.277	.277
	UV	120	1.933	.139	1.656	2.210
		150	2.433	.139	2.156	2.710
		Anti	2.133	.139	1.856	2.410
		Jute	8.135E-15	.139	-.277	.277

Post Hoc Tests

StorageTime

Multiple Comparisons

Dependent Variable:Caraway

	(I) StorageTime	(J) StorageTime	Mean Difference (I-J)	Std. Error	Sig.	95% Confidence Interval Lower Bound	Upper Bound
Tukey HSD	0 time	12 mon	.8600*	.06961	.000	.6657	1.0543
		3 mont	.6700*	.06961	.000	.4757	.8643
		6 mont	.6450*	.06961	.000	.4507	.8393
		9 mont	.7575*	.06961	.000	.5632	.9518
	12 mon	0 time	-.8600*	.06961	.000	-1.0543	-.6657
		3 mont	-.1900	.06961	.058	-.3843	.0043
		6 mont	-.2150*	.06961	.023	-.4093	-.0207
		9 mont	-.1025	.06961	.583	-.2968	.0918
	3 mont	0 time	-.6700*	.06961	.000	-.8643	-.4757
		12 mon	.1900	.06961	.058	-.0043	.3843
		6 mont	-.0250	.06961	.996	-.2193	.1693
		9 mont	.0875	.06961	.718	-.1068	.2818

	6 mont	0 time	-.6450*	.06961	.000	-.8393	-.4507
		12 mon	.2150*	.06961	.023	.0207	.4093
		3 mont	.0250	.06961	.996	-.1693	.2193
		9 mont	.1125	.06961	.492	-.0818	.3068
	9 mont	0 time	-.7575*	.06961	.000	-.9518	-.5632
		12 mon	.1025	.06961	.583	-.0918	.2968
		3 mont	-.0875	.06961	.718	-.2818	.1068
		6 mont	-.1125	.06961	.492	-.3068	.0818

Based on observed means.

The error term is Mean Square(Error) = .058.

*. The mean difference is significant at the .05 level.

Homogeneous Subsets

Caraway

	StorageTime	N	Subset		
			1	2	3
Tukey HSD[a]	12 mon	24	1.3933		
	9 mont	24	1.4958	1.4958	
	3 mont	24	1.5833	1.5833	
	6 mont	24		1.6083	
	0 time	24			2.2533
	Sig.		.058	.492	1.000
Duncan[a]	12 mon	24	1.3933		
	9 mont	24	1.4958	1.4958	
	3 mont	24		1.5833	
	6 mont	24		1.6083	
	0 time	24			2.2533
	Sig.		.145	.131	1.000

Means for groups in homogeneous subsets are displayed.

Based on observed means.

The error term is Mean Square(Error) = .058.

a. Uses Harmonic Mean Sample Size = 24.000.

PackagingType

Multiple Comparisons

Dependent Variable:Caraway

	(I) PackagingType	(J) PackagingType	Mean Difference (I-J)	Std. Error	Sig.	95% Confidence Interval Lower Bound	Upper Bound
Tukey HSD	120	150	-.1287	.06226	.173	-.2920	.0347
		Anti	-.0147	.06226	.995	-.1780	.1487
		Jute	.7533*	.06226	.000	.5900	.9167
	150	120	.1287	.06226	.173	-.0347	.2920
		Anti	.1140	.06226	.267	-.0494	.2774
		Jute	.8820*	.06226	.000	.7186	1.0454
	Anti	120	.0147	.06226	.995	-.1487	.1780
		150	-.1140	.06226	.267	-.2774	.0494
		Jute	.7680*	.06226	.000	.6046	.9314
	Jute	120	-.7533*	.06226	.000	-.9167	-.5900
		150	-.8820*	.06226	.000	-1.0454	-.7186
		Anti	-.7680*	.06226	.000	-.9314	-.6046

Based on observed means.

The error term is Mean Square(Error) = .058.

*. The mean difference is significant at the .05 level.

Homogeneous Subsets

Caraway

	PackagingType	N	Subset 1	2
Tukey HSD[a]	Jute	30	1.0660	
	120	30		1.8193
	Anti	30		1.8340
	150	30		1.9480
	Sig.		1.000	.173
Duncan[a]	Jute	30	1.0660	

120	30		1.8193
Anti	30		1.8340
150	30		1.9480
Sig.		1.000	.053

Means for groups in homogeneous subsets are displayed.

Based on observed means.

The error term is Mean Square(Error) = .058.

a. Uses Harmonic Mean Sample Size = 30.000.
